# SNF1‐related protein kinase 1 represses *Arabidopsis* growth through post‐translational modification of E2Fa in response to energy stress

**DOI:** 10.1111/nph.18597

**Published:** 2022-12-07

**Authors:** Seungmin Son, Jong Hee Im, Jae‐Heung Ko, Yeirin Lee, Sang‐Won Lee, Kyung‐Hwan Han

**Affiliations:** ^1^ Department of Life Sciences Korea University 145 Anamro, Sungbuk‐gu Seoul 02841 Korea; ^2^ National Institute of Agricultural Sciences, Rural Development Administration Jeonju 54874 Korea; ^3^ Department of Horticulture Michigan State University East Lansing MI 48824 USA; ^4^ Department of Plant & Environmental New Resources, College of Life Science and Graduate School of Biotechnology Kyung Hee University Yongin‐si Gyeonggi‐do 17104 Korea; ^5^ Department of Chemistry, Center for Proteogenome Research Korea University 145 Anamro, Sungbuk‐gu Seoul 02841 Korea; ^6^ Department of Forestry Michigan State University East Lansing MI 48824 USA

**Keywords:** *Arabidopsis thaliana*, cell cycle, E2Fa, energy stress, protein degradation, protein phosphorylation, SnRK1

## Abstract

- Cellular sugar starvation and/or energy deprivation serves as an important signaling cue for the live cells to trigger the necessary stress adaptation response. When exposed to cellular energy stress (ES) conditions, the plants reconfigure metabolic pathways and rebalance energy status while restricting vegetative organ growth. Despite the vital importance of this ES‐induced growth restriction, the regulatory mechanism underlying the response remains largely elusive in plants.
- Using plant cell‐ and whole plant‐based functional analyses coupled with extended genetic validation, we show that cellular ES‐activated SNF1‐related protein kinase 1 (SnRK1.1) directly interacts with and phosphorylates E2Fa transcription factor, a critical cell cycle regulator.
- Phosphorylation of E2Fa by SnRK1.1 leads to its proteasome‐mediated protein degradation, resulting in S‐phase repression and organ growth restriction.
- Our findings show that ES‐dependently activated SnRK1.1 adjusts cell proliferation and vegetative growth for plants to cope with constantly fluctuating environments.

## Introduction

Plants have integrated developmental, metabolic, and physiological responses to survive unfavorable growth conditions (e.g. abiotic stress, limited light, or nutrient). Cellular energy stress (ES) conditions serve as a signaling cue to trigger such stress adaptation processes, leading to metabolite redistribution to quiescent meristems and/or storage organs while restraining vegetative organ growth (Rolland *et al*., 2006; Zhu, 2016; Crepin & Rolland, 2019). For example, most land plants grow slowly and eventually terminate vegetative growth under low oxygen and extended darkness (Cho *et al*., 2012; Lim *et al*., 2021). Despite the vital importance of the plant's ability to adapt and sustain under cellular ES conditions, cellular signaling and regulatory mechanisms in the stress adaptation process remain largely elusive.

Cell proliferation is a key determinant of organismal growth, development, and patterning (Komaki & Sugimoto, 2012; Scofield *et al*., 2014). Regulatory modules for cell cycle‐dependent proliferation are widely conserved in most eukaryotes, including plants. Evolutionarily conserved genes encoding for cell cycle regulators are found in a diverse array of plant genomes (Harashima *et al*., 2013; Liu *et al*., 2018). The *Arabidopsis thaliana* genome contains several key transcription factors of cell cycle regulatory modules (e.g. E2Fa, E2Fb, and E2Fc) that have high sequence similarities to those in many other species (Vandepoele *et al*., 2002; Magyar *et al*., 2005). E2Fa and E2Fb are transcription activators responsible for S‐phase‐specific gene expression, contributing to DNA replication, DNA repair, and chromatin maintenance (Ramirez‐Parra *et al*., 2003; Vandepoele *et al*., 2005; Takahashi *et al*., 2008; Leviczky *et al*., 2019). In addition, certain plant hormones have been known for their function in the G1‐to‐S transition during plant cell division. For example, the plant stress hormone abscisic acid (ABA) reduces CDKA (cyclin‐dependent kinase A) level (Garza‐Aguilar *et al*., 2017), while another plant stress and defense hormone, ethylene, suppresses CDKA activity, resulting in plant growth suppression (Skirycz *et al*., 2011).

During cellular energy starvation, yeast SUCROSE NON‐FERMENTING1 (SNF1), animal AMP‐ACTIVATED PROTEIN KINASE (AMPK), and plant SNF1‐RELATED PROTEIN KINASE1 (SnRK1) function as evolutionarily conserved energy sensors (Polge & Thomas, 2007; Hedbacker & Carlson, 2008; Narbonne & Roy, 2009). SnRK1.1/AKIN10 was first identified for its ability to complement yeast *snf1* (Alderson *et al*., 1991). SnRK1.1 and SnRK1.2 serve as two functionally redundant α‐subunits of plant SnRK1 complex in *Arabidopsis* (Baena‐Gonzalez *et al*., 2007). Like AMPK and SNF1, SnRK1 plays fundamental roles in plant's response to cellular ES, such as low oxygen and darkness (Halford & Hey, 2009; Cho *et al*., 2016).

Under cellular energy starvation conditions, phosphorylation of the C‐group bZIP transcription factor bZIP63 by SnRK1.1 plays a role in the transcriptional reconfiguration of metabolic pathway and reduces dark‐induced senescence (Mair *et al*., 2015). Nuclear localization of SnRK1.1 is rhythmic and plays a role in not only metabolic stress responses but also organ growth and development (Yuan *et al*., 2016; Ramon *et al*., 2019). Thus, a better understanding of the nuclear activity of SnRK1.1 promises to yield deeper insights into how the ES‐inducible protein kinase activity would contribute to life‐sustaining processes in plant cells under unfavorable environmental conditions.

Overexpression of SnRK1 kinase correlates well with organ growth restriction (Tomé *et al*., 2014; Broeckx *et al*., 2016; Hulsmans *et al*., 2016). *Arabidopsis* SnRK1 activation leads to growth restriction and organ senescence delay (Baena‐Gonzalez *et al*., 2007; Cho *et al*., 2012; Tsai & Gazzarrini, 2012). Conversely, increased levels of trehalose‐6‐phosphate (T6P), an SnRK1 inhibitor, lead to plant growth promotion (Zhang *et al*., 2009; Paul *et al*., 2010; Debast *et al*., 2011) while decreased T6P levels suppress the expression of various cell proliferation‐related genes in transgenic potato (*Solanum tuberosum*) plants (Debast *et al*., 2011). However, the biochemical and molecular mechanisms underlying this SnRK1‐mediated cell cycle regulation remain unclear, prompting our interest in the role of SnRK1.1 kinase and ES signaling in plant growth restriction via direct cell cycle regulation. We hypothesized that the ES‐inducible kinase SnRK1.1 negatively regulates cell proliferation and plant growth through post‐translational modification (i.e. phosphorylation) of E2Fa, a key transcription activator responsible for S‐phase‐specific gene expression (Vandepoele *et al*., 2002, 2005; Ramirez‐Parra *et al*., 2003; Magyar *et al*., 2005; Takahashi *et al*., 2008; Kállai *et al*., 2020).

Here, we report that SnRK1.1 directly interacts with and phosphorylates the marked box (MB) domain (Mariconti *et al*., 2002) of the transcription factor E2Fa. The SnRK1.1‐mediated phosphorylation of E2Fa leads to proteasome‐dependent protein degradation of E2Fa, resulting in downregulation of S‐phase‐specific gene expression and cell cycle arrest under ES conditions. Our findings reveal that a cellular energy sensor SnRK1.1 serves as a key regulatory element in the control of cell proliferation for plant survival and adaptation to unfavorable growth conditions.

## Materials and Methods

### Plant materials and growth conditions

*Arabidopsis thaliana* Columbia ecotype (Col‐0) was used as the wild‐type (WT) plant in this study. Transgenic *Arabidopsis* Col‐0 plants expressing *SnRK1.1* ^*WT*^, *SnRK1.1* ^*IN*^, *pE2Fa::gE2Fa‐GFP*, *E2Fa RNAi*, and a mutant *e2fa* line were obtained from the published resources (Cho *et al*., 2012; Magyar *et al*., 2012; Xiong *et al*., 2013). The *pE2Fa::gE2Fa‐GFP/SnRK1.1* ^*WT*^ and *pE2Fa::gE2Fa‐GFP/ SnRK1.1* ^*IN*^ lines were generated through genetic crosses.

For *e2fa* complementary line generation, the promoter of *E2Fa* (*pE2Fa*) was PCR‐amplified and substituted with the *35S* promoter in the pBI121 binary vector (*pE2Fa*_pBI121). A full‐length *E2Fa* cDNA was PCR‐amplified from Col‐0 cDNA and cloned into the C‐terminal 2x*MYC*‐tagged HBT vector. The *E2Fa‐2xMYC2‐NOS* terminator cassette from the HBT vector was fused to the *pE2Fa*_pBI121 vector by restriction enzyme digestion and ligation (*pE2Fa*::*E2Fa*‐2x*MYC2*). The *E2Fa* ^*T314AT315A*^ was generated by site‐direct mutagenesis and cloned into the *2xHA*‐tagged HBT vector. The *E2Fa* ^*T314AT315A*^ *‐2xHA‐NOS* terminator cassette from the HBT vector was fused to the pE2Fa_pBI121 vector by restriction enzyme digestion and ligation. These constructs were transformed into the *e2fa* mutant line. The transgenic plants were selected as single‐insertion homozygous lines.

*Arabidopsis* seeds were grown either in six‐well plates (six seeds per well) containing 1 ml of ½ Murashige & Skoog (½MS) liquid medium (0.5% sucrose, pH 5.7 adjusted with KOH) or in soil for indicated days in a growth room at 23°C, 50% humidity, and 75 μmol m^−2^ s^−1^ light intensity under 16 h : 8 h, light : dark photoperiod.

### Energy stress and phytohormone treatments

Four‐day‐old *Arabidopsis* seedlings were transferred to a hypoxic chamber with 1% O_2_, < 0.1% CO_2_, and 98.9% N_2_ (Ruskinns Invivo_300_; Ruskinn Technology Ltd, Bridgend, UK) in submerged and dark conditions with and without carbon supplementation. For phytohormone treatment, 4‐d‐old seedlings were transferred to ½MS liquid medium (0.5% sucrose, pH 5.7 adjusted with KOH) containing 100 μM ABA, ACC, or MeJA, and then incubated for 6 h.

### EdU‐based S‐phase assay

5‐ethynyl‐2′‐deoxyuridine (EdU) staining was performed using the Click‐iT EdU Fluor 488 kit (Thermo Fisher Scientific, Waltman, MA, USA) as described previously (Kotogany *et al*., 2010; Xiong *et al*., 2013). The samples were imaged using an LSM700 confocal laser‐scanning microscope (Carl Zeiss).

### Transient promoter assay in mesophyll protoplasts

*Arabidopsis* mesophyll protoplast transient promoter assays were performed as described previously (Yoo *et al*., 2007). To generate effector constructs, full‐length cDNA of *SnRK1.1* ^*WT*^, *SnRK1.1* ^*IN*^, *E2Fa*, *E2Fb*, and *E2Fc* was PCR‐amplified and cloned into the *35SC4PPDK* promoter and the *NOS* terminator in plant cell expression vector, *pHBT*. *E2Fa* ^*S93A*^, *E2Fa* ^*T99A*^, *E2Fa* ^*S170A*^, *E2Fa* ^*S278A*^, *E2Fa* ^*T314AT315A*^, *E2Fa* ^*S427A*^, and *E2Fa* ^*S454A*^ were generated with site‐direct mutagenesis. SPYNE (the N‐terminal end of split YFP) was used as effector control. To generate promoter reporter, the *35SC4PPDK* promoter of *pHBT‐fLUC* (*firefly luciferase*) was replaced by target promoter. For protein translation reporter, full‐length cDNA of target protein cloned into the *35SC4PPDK* promoter and *fLUC* in *pHBT‐fLUC*. The *35S*‐driven *rLUC* (*renilla luciferase*) in *pHBT* was used as an expression control. A total of 40 μg of the desired DNA constructs were transfected to protoplasts (6 × 10^4^ cells per 200 μl) by the PEG method and incubated in WI solution for 6 h at 25°C. For chemical treatment, DMSO or 5 μM MG132 was added to the solution after 1 h of incubation. The experiments were independently carried out at least three times, and the data were analyzed by *t*‐test.

### Co‐immunoprecipitation

Co‐immunoprecipitation (Co‐IP) was performed as described previously (Xiong *et al*., 2013) with slight modifications. Briefly, 300 μg of each *E2Fa‐GFP* was transfected with or without 100 μg of *SnRK1.1‐HA* to 2 ml of protoplasts (4.5 × 10^5^ cells) and incubated 11 h in W5 solution containing 5 μM MG132. After washing with WI solution, transfected protoplasts were lysed in 200 μl of lysis buffer. The samples were then spun down, and the supernatant was transferred into 2‐ml tubes. After the transfer of 20 μl of the sample as an input fraction, the volume of the lysate‐supernatants was adjusted to 500 μl with dilution buffer. Twenty‐five microliters of bead slurry (ChromoTek, Planegg, Germany) was added and incubated for 6 h on shaker at a 4°C. After the beads were washed three times with 500 μl ice‐cold wash buffer, the samples were resuspended with 50 μl of 2× sample buffer. The presence of co‐immunoprecipitated proteins was detected by immunoblotting.

### Protein immunoblot analysis

Total proteins were extracted using extraction buffer (50 mM Tris‐Base, 150 mM NaCl, 10 mM NaF, 10 mM Na3Vo4, 1× protease inhibitor cocktail, and 0.2% (v/v) Triton X‐100). After centrifugation at 16 000 ***g*** for 10 min at 4°C, the supernatants were collected into new tubes. After protein concentrations were measured by the Bio‐Rad protein assay, the total proteins in the supernatants were separated by sodium dodecyl sulfate–polyacrylamide gel electrophoresis (SDS‐PAGE) and then transferred to polyvinylidene difluoride membranes. For immunoblotting, primary antibodies such as anti‐MYC (Roche), anti‐HA (Roche), anti‐GFP (Clontech, Mountain View, CA, USA), anti‐GST (Cell signaling, Danvers, MA, USA), anti‐SnRK1.1 (Agrisera, Vannas, Sweden), anti‐pT172‐AMPKα antibody (Cell Signaling), and anti‐Actin11 (Agrisera) were used (1 : 1000). Infrared‐800‐conjugated secondary antibody was added (1 : 10 000). The signal was detected using an IR‐image detector Odyssey (Li‐Cor, Lincoln, NE, USA), and quantitative analysis was carried out with ImageJ.

### *In vitro* kinase assay

To prepare the kinase and substrates, each cDNA of *E2Fa*, *E2FaMB*, *E2FaMB* ^*T314AT315A*^, *E2FaMB* ^*S338AT339A*^, *E2FaMB* ^*T314AT315AS338AT339A*^, *E2Fb*, *E2Fc*, *GRIK1*, and *SnRK1.1* was fused to the C‐terminal end of *GST* in the *pGEX* vector. N‐terminal GST‐tagged recombinant E2Fa, E2FaMB, E2FaMB^T314AT315A^, E2FaMB^S338AT339A^, and E2Fc proteins were expressed in *Escherichia coli* strain BL21(DE3) or BL21(DE3) pLysS in the presence of 1 mM IPTG at 28°C. N‐terminal GST‐tagged recombinant E2Fb, GRIK1, and SnRK1.1 proteins were expressed in Rosetta (DE3) in the presence of 1 mM IPTG at 28°C. Total proteins were extracted after sonication and affinity‐purified using Glutathione Sepharose 4B (GE Healthcare, Chicago, IL, USA). For SnRK1.1 kinase assay, GFP‐ or HA‐tagged SnRK1.1 was transiently expressed in mesophyll protoplasts. The cells were lysed completely with kinase‐lysis buffer (20 mM Tris (pH 7.5), 40 mM MgCl_2_, 5 mM EDTA, 1 mM DTT, 10 mM NaF, 10 mM Na_3_Vo_4_, 1× protease inhibitor cocktail, and 1% Triton X‐100). For immunoprecipitation (IP) analysis of GFP‐ or HA‐tagged SnRK1.1, protein extracts were incubated with 1.5 μl tag‐specific antibody at 4°C for 3 h and an additional 3 h after adding 10 μl A‐agarose beads. The beads were washed with IP buffer (50 mM Tris (pH 7.5), 150 mM NaCl, 5 mM EDTA, 1 mM DTT, and 1× protease inhibitor cocktail) and then twice with kinase buffer (20 mM Tris (pH 7.5), 40 mM MgCl_2_, 5 mM EDTA, and 1 mM DTT). Kinase reaction was performed with radioactive ATP as described previously (Im & Yoo, 2014).

### Yeast two‐hybrid assay

Yeast two‐hybrid assay was performed using yeast two‐hybrid system (Clontech). Each cDNA of *SnRK1.1*, *SnRK1.1* ^*1–396*^, *SnRK1.1* ^*397–512*^ was cloned into *pGBKT7*. *E2Fa*, *E2Fa* ^*T314DT315D*^, *E2Fa* ^*1–281*^, *E2Fa* ^*282–352*^, *E2Fa* ^*353–485*^, and *E2Fb* were cloned into *pGADT7* vectors. The constructs were co‐transformed with designated combination to the yeast (AH109), and double transformants were selected as cells grown in the media lacking leucine and tryptophan. The selected double transformants were transferred and grown in the media lacking histidine, leucine, and tryptophan with 0.5 mM 3‐amino‐1,2,4‐triazole. For the serial dilution assay, yeast cells were harvested and adjusted to an OD_600_ of 0.5 with sterilized double‐distilled water and diluted to 1/10, 1/100, and 1/1000. A positive control was carried out with *pGBKT7‐53* and *pGADT7‐T* constructs, and a negative mating was carried out with *pGBKT7‐Lam* and *pGADT7‐T* constructs.

### Gene expression analysis

To analyze gene expression, total RNA was isolated from plants using the TRIzol reagent (Invitrogen), and 1 μg of total RNA was used for cDNA synthesis using M‐MLV reverse transcriptase (Promega). Reverse transcription polymerase chain reaction was performed using CFX96 Real‐Time System (Bio‐Rad) with gene‐specific primers listed in Table S1. The *UBQ10* and *EIF4a* genes were used as expression controls.

### Cell‐free degradation assay

Col‐0 and *SnRK1.1* ^*WT*^ seedlings were ground in liquid nitrogen with protein stability assay buffer (25 mM Tris–HCl (pH 7.5), 10 mM NaCl, 10 mM MgCl_2_, 5 mM DTT, 10 mM ATP, and 4 mM PMSF) and centrifuged twice at 16 000 ***g*** for 15 min at 4°C, and the supernatants were collected into new tubes. After protein concentrations were measured by the Bio‐Rad protein assay, total protein extracts from Col‐0 and *SnRK1.1* ^*WT*^ transgenic plants were adjusted to equal concentration with protein stability assay buffer. Purified, recombinant GST‐E2Fa and GST‐E2Fa^T314AT315A^ proteins generated in *E. coli* were incubated for indicated time in equal quantities of plant extracts with DMSO or 40 μM MG132 at 22°C, and the reaction was stopped by 5× SDS‐PAGE sample buffer. An equal volume of each sample was separated in 8% SDS‐PAGE gel, and immunoblots were performed.

### Protein mobility shift assay

Protein mobility shift assay was performed as described previously with slight modification (Im *et al*., 2014). For the protein mobility shift assay, E2Fa‐MYC and E2Fa^T314AT315A^‐MYC were expressed in *Arabidopsis* protoplasts with 10 μM MG132 with or without SnRK1.1^WT^, then separated on a 10% (w/v) SDS‐PAGE for 2 h, and identified by protein blot analysis using anti‐MYC antibody.

### In‐gel digestion

In‐gel digestion was performed using a modified protocol of a previously reported method (Shevchenko *et al*., 2007). *In vitro* kinase assay was carried out with SnRK1.1 and E2Fa and separated the proteins with PAGE. A band of E2Fa was sliced into small pieces (*c*. 1 × 1 mm^2^) with a clean blade, and the gel pieces were transferred into a microcentrifuge tube. The samples were used for the analysis of the phosphorylation site of E2Fa by LC–MS/MS.

### Statistical analysis

All experiments were independently conducted at least three times, and the data were analyzed by *t*‐test or ANOVA. Asterisks denote significant differences (**, *P* < 0.01; *, *P* < 0.05), and different letters indicate statistical differences (*P* < 0.05).

## Results

### Cellular energy deprivation induces organ growth restriction

With the cellular ES treatment of low oxygen (< 1%) and extended darkness that interfere normal photosynthesis (Fig. S1a), the growth of rosettes, cotyledons, and primary roots was reduced (Fig. S1b,c). The ES‐induced growth reduction was reversed when sucrose was added to the medium (Fig. S1c).

To examine whether the cellular ES‐induced growth restriction was due to a lack of cell division, S‐phase cell division activity in the primary root meristems was monitored by EdU staining, which detects DNA synthesis in proliferating cells (Xiong *et al*., 2013). EdUfluorescence was detected in the cell division zone of the primary roots but was abolished following ES treatment (Fig. S1d), indicating that cell division was stopped with the ES treatment.

We then examined the expression of several marker genes related to cellular ES and S‐phase activity in *Arabidopsis* seedlings. The expression of two SnRK1.1‐activated genes *DARK INDUCIBLE 1* (*DIN1*) and *DIN6* (Baena‐Gonzalez *et al*., 2007) was significantly increased by the ES treatment (Fig. S1e), while the expression of two key S‐phase‐specific transcription activators *E2Fa* and *E2Fb* was slightly reduced to about 70–80% by the ES treatment, compared with the control plants (Fig. S1f). However, the expression of the direct target genes of E2Fs was decreased markedly (Fig. S1f), suggesting that ES restricts plant growth through negative regulation of S‐phase activity.

### SnRK1.1 is activated by energy stress and restricts organ growth

**Fig. 1:**
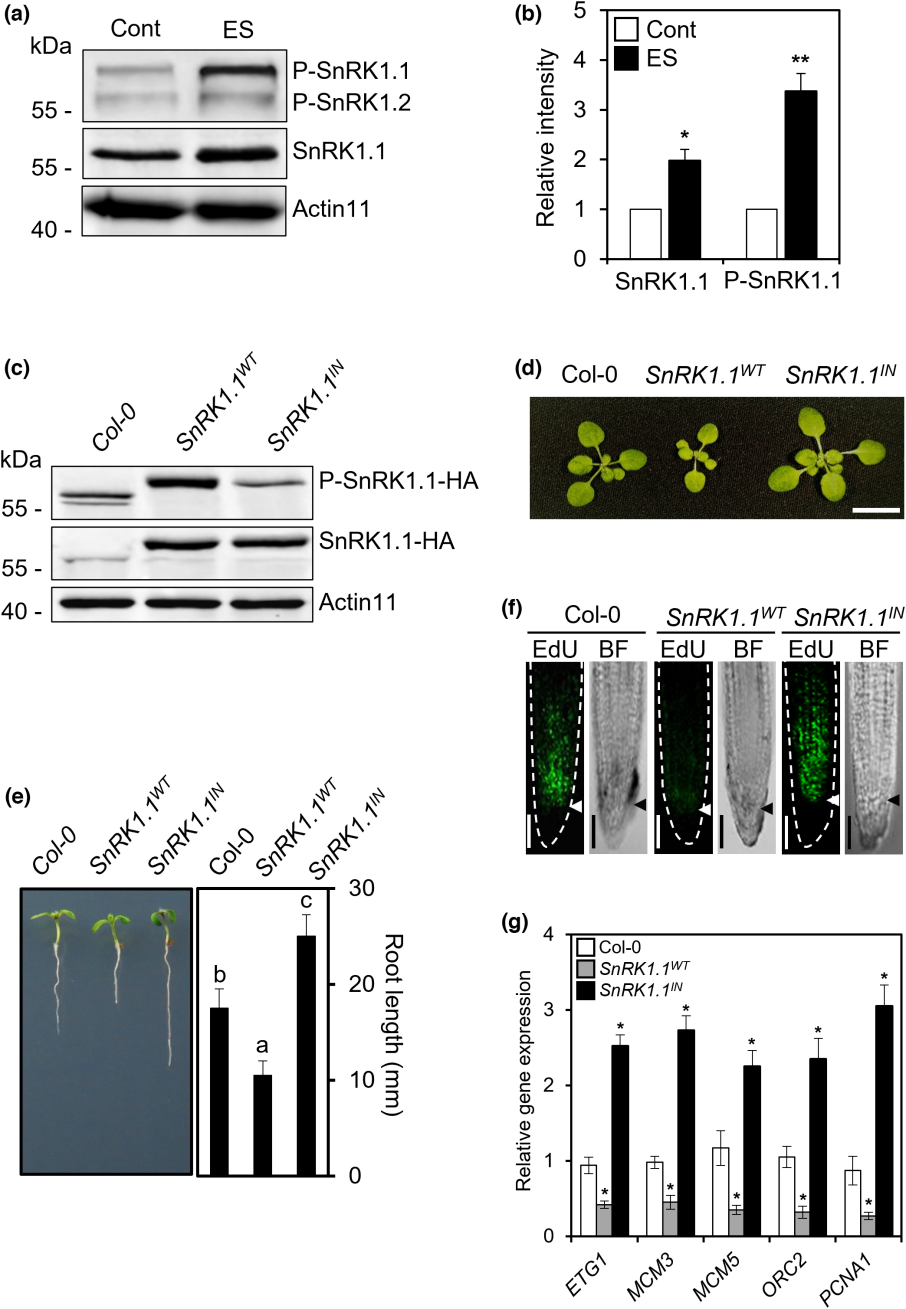
Cellular energy stress (ES) induces organ growth restriction in *Arabidopsis*. (a, b) Protein blot (a) and quantitative (b) analysis of total SnRK1.1 and phosphorylated SnRKs (P‐SnRK1.1 and P‐SnRK1.2) in Col‐0 after ES treatment for 24 h on 4‐d‐old seedlings grown in ½MS liquid medium containing 0.5% sucrose. Total protein from the plants was used in the protein blot analysis with anti‐pT172‐AMPKα antibody for the detection of phosphorylated SnRKs and anti‐SnRK1.1 for protein level of SnRK1.1. Actin11 was used as a control. Values are mean ± SD. Asterisks indicate values statistically different from controls (*t*‐test; **, *P* < 0.01; *, *P* < 0.05). (c) Protein blot analysis of SnRK1.1 and phosphorylated SnRK1.1 (P‐SnRK1.1) of 5‐d‐old Col‐0, transgenic plants expressing *SnRK1.1* ^*WT*^ and *SnRK1.1* ^*IN*^. Total protein from the plants was used in the protein blot analysis with anti‐pT172‐AMPKα antibody and anti‐HA antibody. Actin11 was used as a control. (d) Growth phenotypes of 3‐wk‐old Col‐0 and *SnRK1.1* ^*WT*^‐, or *SnRK1.1* ^*IN*^‐expressing transgenic plants grown on soil. Bar, 20 mm. (e) Primary root growth and quantitative analysis of Col‐0 plants expressing *SnRK1.1* ^*WT*^ and *SnRK1.1* ^*IN*^. The root length of 5‐d‐old plants grown in ½MS liquid medium containing 0.5% sucrose was measured. Values are mean ± SD. Different letters indicate statistical differences according to ANOVA (*P* < 0.05). All experiments were repeated at least three times with similar results. (f) Cellular images of EdU staining of the root apical meristems of 5‐d‐old Col‐0 and *SnRK1.1* ^*WT*^ or *SnRK1.1* ^*IN*^‐expressing transgenic plants grown in ½MS liquid medium containing 0.5% sucrose. Bright‐field images served as a control. Arrowheads indicate quiescent center. BF, bright field; Bar, 100 μm. (g) Expression analysis of cell cycle‐related genes of 5‐d‐old Col‐0 and *SnRK1.1* ^*WT*^ or *SnRK1.1* ^*IN*^‐expressing transgenic plants using RT‐qPCR. The plants were grown in ½MS liquid medium containing 0.5% sucrose for 5 d, and total RNA was isolated from the plants and synthesized cDNA with the RNA. *EIF4a* was used as a control. Values are mean ± SD. Asterisks indicate values statistically different from Col‐0 control (*t*‐test; *, *P* < 0.05).

Given that SnRK1 plays a fundamental role in plant response to cellular ES (Hulsmans *et al*., 2016) and the expression of SnRK1.1‐inducible *DIN1* and *DIN6* was significantly increased by ES treatment (Fig. S1e), we reasoned that ES may activate SnRK1.1 kinase activity. To test this hypothesis, we first analyzed SnRK1.1 protein accumulation using anti‐SnRK1.1 antibodies and then measured T‐loop‐phosphorylated SnRK1.1 (P‐SnRK1.1; i.e. its activation) using anti‐pT172‐AMPKα antibodies (Crozet *et al*., 2014). Both SnRK1.1 and P‐SnRK1.1 protein levels were increased compared with those of Actin11 by the cellular ES treatment (Fig. 1a,b).

We further hypothesized that increased activity of SnRK1.1 may lead to growth restriction through the inhibition of S‐phase. To test this hypothesis, we used transgenic *Arabidopsis* plants overexpressing either hemagglutinin (HA) epitope‐tagged normal *SnRK1.1* (*SnRK1.1* ^*WT*^) or inactive form of *SnRK1.1* (*SnRK1.1* ^*IN*^). Since single‐knockout mutants of SnRK1s such as *snrk1.1* and *snrk1.2* did not produce any detectible phenotype, we used *SnRK1.1* ^*IN*^ as a loss‐of‐function variant in this study (Fig. S2). The SnRK1.1^IN^ was created by substituting Lys48 with Met residue to block the ATP‐binding site of the protein kinase (Baena‐Gonzalez *et al*., 2007; Im *et al*., 2014). While both SnRK1.1^WT^‐HA and SnRK1.1^IN^‐HA proteins were clearly detected using anti‐HA antibody in the respective transgenic plants, the level of T‐loop phosphorylated SnRK1.1 was substantially reduced in the SnRK1.1^IN^ plants (Fig. 1c), suggesting that SnRK1.1^IN^ may function as dominant‐negative regulator in the SnRK1 signaling as reported previously (Baena‐Gonzalez *et al*., 2007).

As expected, *SnRK1.1* ^*WT*^ transgenic plants had smaller rosettes than did WT Col‐0 and *SnRK1.1* ^*IN*^ transgenic plants (Fig. 1d). Also, the *SnRK1.1* ^*WT*^ transgenic seedlings had shorter primary roots than the Col‐0 plants (Fig. 1e), suggesting that SnRK1.1 may negatively regulate plant growth. To corroborate this observation, we analyzed DNA synthesis (i.e. cell division) activity in the primary roots using Edu assay. Edu staining was much lower in the *SnRK1.1* ^*WT*^ plants than in Col‐0 and *SnRK1.1* ^*IN*^ transgenic seedlings (Fig. 1f).

To further confirm the SnRK1.1‐mediated cell division repression, the expression of S‐phase‐specific genes *E2F TARGET GENE 1* (*ETG1*), *MINICHROMOSOME MAINTENANCE 3* (*MCM3*), *MCM5*, *ORIGIN RECOGNITION COMPLEX SECOND LARGEST SUBUNIT 2* (*ORC2*), and *PROLIFERATING CELLULAR NUCLEAR ANTIGEN 1* (*PCNA1*) was examined in the Col‐0*, SnRK1.1* ^*WT*^, and *SnRK1.1* ^*IN*^ transgenic seedlings. Their expression was decreased in the *SnRK1.1* ^*WT*^ but significantly increased in the *SnRK1.1* ^*IN*^ transgenic seedlings, compared with their expression in the Col‐0 plants (Fig. 1g). Taken together, SnRK1.1 negatively regulates S‐phase activity in the cell division zone, resulting in growth restriction of *Arabidopsis* seedlings. Moreover, since the inactive form of SnRK1.1 plays a dominant‐negative role with respect to the kinase activity (Baena‐Gonzalez *et al*., 2007; Cho *et al*., 2012), the upregulation of S‐phase‐specific gene expression in the SnRK1.1^IN^ plants supports the hypothesis that SnRK1.1 is a key player in energy starvation‐mediated S‐phase inhibition, hence growth restriction.

### SnRK1.1 represses G1‐S transition

Given that SnRK1.1 negatively regulates S‐phase activity and is known to regulate transcription factors, we asked whether a key cell cycle regulator E2Fa is involved in the SnRK1.1‐mediated S‐phase suppression. To genetically test this, we investigated the growth of cotyledons and primary roots, which are under the control of cell division, in various SnRK1.1 and E2Fa lines (Cho *et al*., 2012; Magyar *et al*., 2012; Xiong *et al*., 2013). The *SnRK1.1* ^*WT*^, *e2fa*, and *E2Fa RNAi* transgenic plants had shorter primary root length and smaller cotyledon than the WT Col‐0 plants (Fig. S3a). However, the cotyledons and primary roots of *SnRK1.1* ^*IN*^ and *pE2Fa::gE2Fa‐GFP* transgenic plants (i.e. genetic complementation of *e2fa*) were larger and longer, respectively, than those of the *SnRK1.1* ^*WT*^ plants. In light of the fact that SnRK1.1 negatively regulates both organ development and ES‐induced senescence and inflorescence development (Baena‐Gonzalez *et al*., 2007; Cho *et al*., 2012), we asked whether E2Fa is also involved in the SnRK1.1‐mediated control of inflorescence development and senescence. While E2Fa positively regulated both cotyledon size and root length, it did not have an impact on the development of inflorescence and senescence (Fig. S3b), suggesting E2Fa is involved in SnRK1.1 signaling related to plant growth but not plant development.

### E2Fa protein is negatively regulated under energy stress condition

**Fig. 2:**
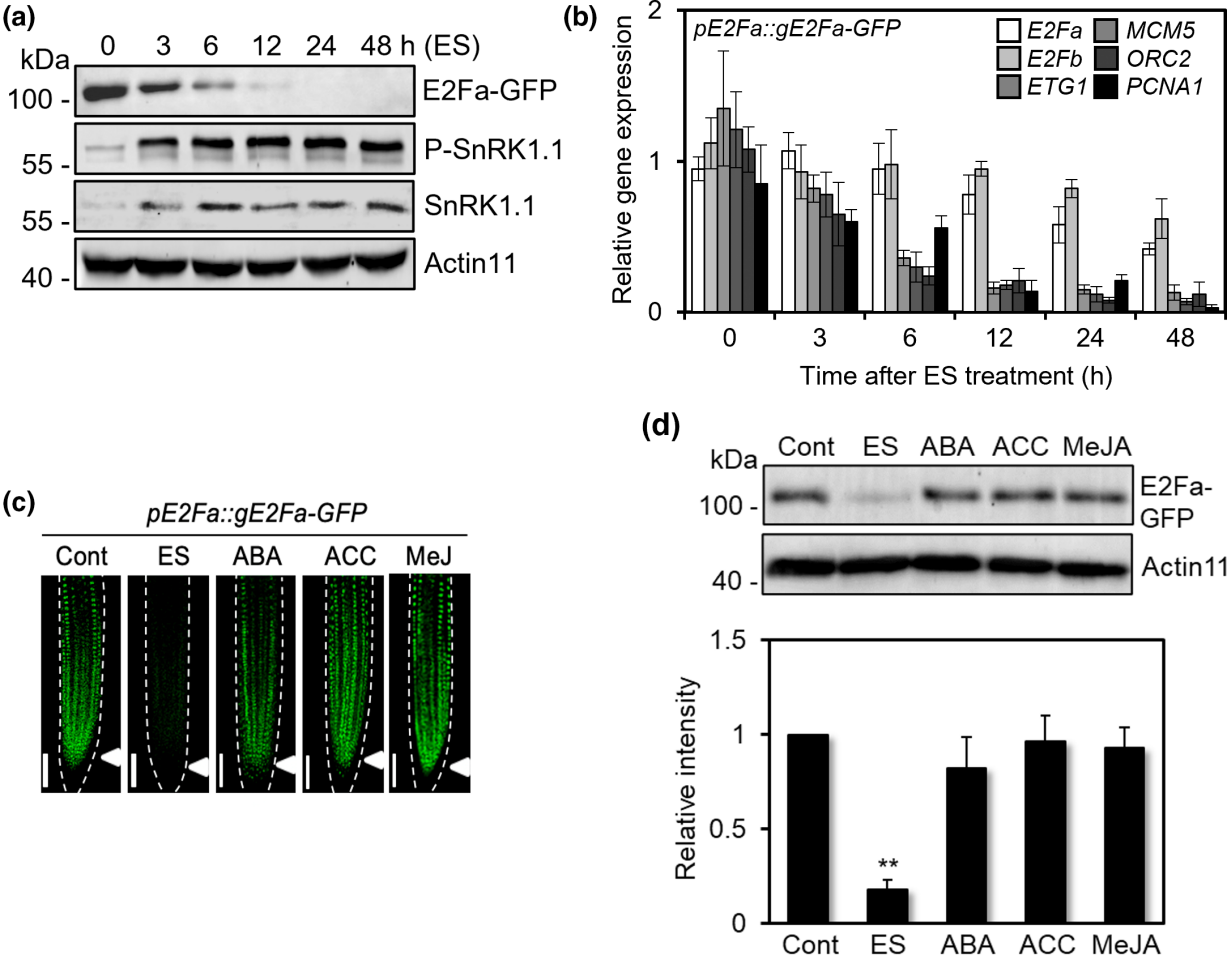
E2Fa activity is post‐translationally regulated under energy stress (ES) conditions. (a) Protein blot analysis of E2Fa, total SnRK1.1, and P‐SnRK1.1 in *pE2Fa::gE2Fa‐GFP* transgenic seedlings (4 d old) after ES treatment for the indicated times. Anti‐GFP (for detection of E2Fa‐GFP), anti‐P‐AMPKα (for the detection of phosphorylated SnRK1.1), and anti‐SnRK1.1 antibodies were used. Actin11 served as a control. (b) Expression analysis of cell cycle‐related genes in *pE2Fa::gE2Fa‐GFP* transgenic seedlings (4‐d‐old) after ES treatment for the indicated hours using RT‐qPCR. *EIF4a* served as a control. Values are mean ± SD. (c) Cellular images of E2Fa‐GFP in root tips of *pE2Fa::gE2Fa‐GFP* transgenic plants after ES, 100 μM ABA, ACC and MeJA treatments on the 4‐d‐old seedling for 6 h. Arrowheads indicate quiescent center. Bar, 100 μm. (d) Protein blot and quantitative analysis of E2Fa‐GFP in *pE2Fa::gE2Fa‐GFP* transgenic seedlings (4 d old) after ES, 100 μM ABA, ACC, and MeJA treatments for 6 h. Actin11 served as a control. Values are mean ± SD. Asterisks indicate values statistically different from control (*t*‐test; **, *P* < 0.01). [Correction added after online publication 7 December 2022: label in panel (a) has been updated.]

Based on the observation that the ES treatment eliminated the expression of *E2Fa* target genes, albeit the expression of *E2Fa* gene itself was very slightly reduced by the treatment (Fig. S1f), we hypothesized that E2Fa activity might be post‐translationally regulated under ES conditions. To test this hypothesis, we investigated E2Fa protein stability using transgenic plants expressing *green fluorescence protein* (*GFP*) fused to the C‐terminus of *E2Fa* gene under the control of own promoter (*pE2Fa::gE2Fa‐GFP*). With ES treatment, E2Fa‐GFP protein was rapidly decreased while P‐SnRK1.1 and SnRK1.1 protein levels were increased (Fig. 2a). Consistent with the observed instability of E2Fa protein, the expression of its direct target genes (*ETG1*, *MCM5*, *ORC2*, and *PCNA1*) started to decrease after 6 h of the ES treatment (Fig. 2b), suggesting that ES may negatively regulate E2Fa transcriptional activity.

To investigate whether stress‐related hormone signaling is involved in the ES‐mediated degradation of E2Fa protein, we analyzed E2Fa protein stability in response to ES and plant stress hormone treatments (ABA, ACC, and MeJA). The hormone treatments were applied for 6 h, at which time point most of E2Fa protein is degraded and its target gene expression diminishes by ES treatment (Fig. 2a,b). The fluorescence signal of the E2Fa‐GFP was abolished by ES, but not by the stress hormones (Fig. 2c). Likewise, E2Fa protein level was reduced significantly by ES, while no change was observed by ABA, ACC, or MeJA treatment (Fig. 2d). These results suggest that, unlike ES, the stress‐related hormones do not affect E2Fa protein level.

### SnRK1.1 suppresses E2Fa‐ and E2Fb‐inducible S‐phase‐specific gene expression

**Fig. 3:**
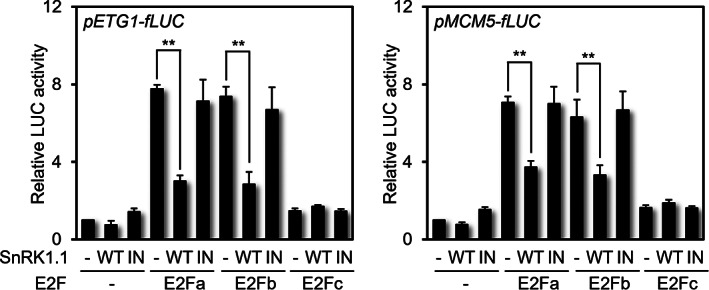
Transcriptional activity of E2Fa/b is negatively regulated by SnRK1.1 Measurements of E2Fa‐, E2Fb‐, and E2Fc‐induced promoter activities of *MCM5* and *ETG1* using *pMCM5‐fLUC* and *pETG1‐fLUC* reporters with combination of SnRK1.1^WT^ and SnRK1.1^IN^ co‐expression. C‐terminal *fLUC*‐conjugated *MCM5* and *ETG1* were co‐expressed in the protoplasts with designated effectors. *pUBQ10‐rLUC* served as an expression control. Values are mean ± SD. Asterisks indicate values statistically different from control (*t*‐test; **, *P* < 0.01).

As a part of the functional analysis of SnRK1.1 kinase in cell cycle‐related gene regulation, we used *fLUC* reporter gene under the control of either S‐phase‐specific *ETG1* or *MCM5* promoter (*pETG1‐fLUC* or *pMCM5‐fLUC*) to analyze E2Fa‐, E2Fb‐, and E2Fc‐dependent reporter fLUC activities in *Arabidopsis* protoplasts. These reporter constructs were transfected to protoplasts with E2Fs, SnRK1.1^WT^, or SnRK1.1^IN^ as effector. The promoter activity was increased with E2Fa or E2Fb expression but repressed significantly by SnRK1.1^WT^ co‐expression but not by SnRK1.1^IN^ (Fig. 3). However, E2Fc, which is known as transcription repressor, did not affect the activity of the S‐phase reporter (Fig. 3). Taken together, SnRK1.1 kinase activity effectively suppresses E2Fa‐ and E2Fb‐mediated gene expression.

### SnRK1.1 directly interacts with and phosphorylates E2Fa

To evaluate the nature of SnRK1.1 and E2Fa/b interaction, we carried out yeast two‐hybrid (Y2H) analyses that showed an interaction between SnRK1.1 and E2Fa or E2Fb (Fig. S4a). Moreover, the C‐terminal regulatory domain of SnRK1.1 interacted with E2Fa or E2Fb (Fig. S4b), indicating that SnRK1.1 may modulate E2Fa and E2Fb functions through direct protein–protein interaction. We then examined whether SnRK1.1 phosphorylates E2Fa and E2Fb by carrying out *in vitro* SnRK1.1 kinase assay using glutathione S*‐*transferase (GST)‐tagged E2Fa/b as protein substrates. E2Fc was used as a negative control substrate. GST‐SnRK1.1 and its upstream activator GST‐GRIK1were used in the *in vitro* kinase assay. The assay showed that GRIK1‐activated SnRK1.1 could phosphorylate E2Fa and E2Fb but not E2Fc (Fig. S4c), confirming that SnRK1.1 phosphorylates the key cell cycle transcription activator E2Fa and E2Fb. Although E2Fa and E2Fb appear to be redundantly required for cell cycle progression, *E2Fa* is expressed predominantly in primary roots, which largely overlaps with *SnRK1.1* expression (Fig. S4d). For this reason, we focused on SnRK1.1‐E2Fa regulon.

**Fig. 4:**
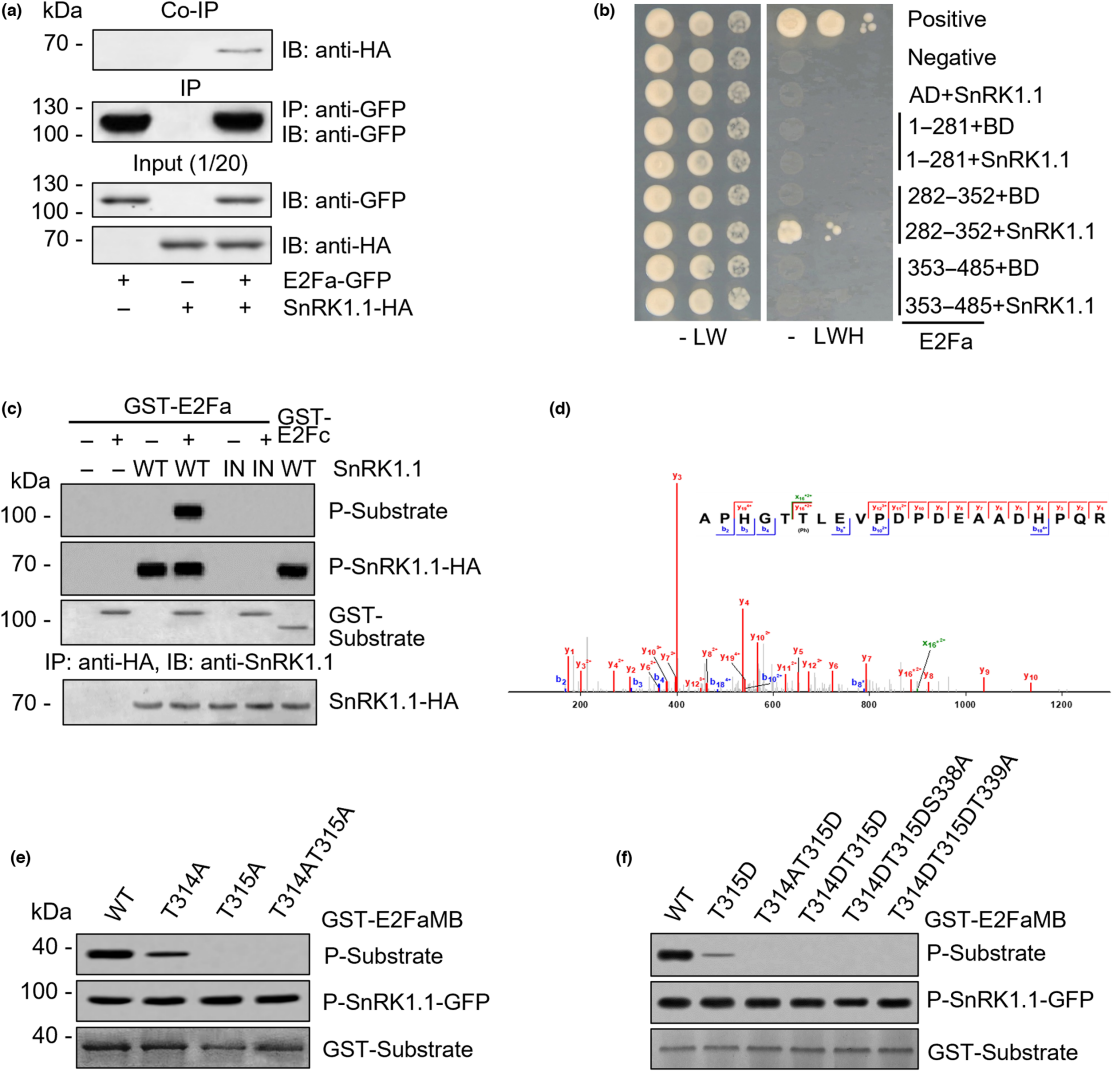
SnRK1.1 directly interacts with and phosphorylates E2Fa. (a) Co‐immunoprecipitation (Co‐IP) for protein–protein interaction between SnRK1.1‐HA and E2Fa‐GFP in protoplast. After the co‐expression of SnRK1.1‐HA and E2Fa‐GFP in the presence of MG132, IP was carried out with anti‐GFP antibody. (b) Yeast two‐hybrid screening of E2Fa domains for SnRK1.1 interaction. After transformation, the yeast cells were grown on selective media lacking Leu and Trp (LW). The yeast cells were additionally selected in selection media (‐LWH) containing 0.5 mM 3‐amino‐1,2,4‐triazole (3‐AT). (c) SnRK1.1 kinase assay with GST‐E2Fa as a protein substrate using P^32^‐[γ]‐ATP. GST‐E2Fc served as a negative control. E2Fs and SnRK1.1 were expressed in *Escherichia coli* and the protoplasts, respectively. *In vitro* kinase assay was carried out using P^32^‐[γ]‐ATP. E2Fc was used as a negative control. (d) Annotated MS/MS spectrum of phosphorylated APHGTTLEVPDPDEAADHPQR. *, Loss of phosphorylation. (e) SnRK1.1 kinase assay with GST‐E2FaMB, GST‐E2FaMB^T314A^, GST‐E2FaMB^T315A^, and GST‐E2FaMB^T314AT315A^ as protein substrates using P^32^‐[γ]‐ATP. GST‐E2FaMB and its designated variants were expressed in *E. coli*, and SnRK1.1 was expressed in protoplasts. *In vitro* kinase assay was carried out using P^32^‐[γ]‐ATP. (f) SnRK1.1 kinase assay for subsequent phosphorylation with GST‐E2FaMB and its designated variants as protein substrates using P^32^‐[γ]‐ATP.

To further ascertain the protein–protein interaction between SnRK1.1 and E2Fa *in vivo*, we carried out Co‐IP analyses in *Arabidopsis* protoplasts. HA‐tagged SnRK1.1 was detected in the immunoprecipitated complex with E2Fa‐GFP, but not in the control (Figs 4a, S5), confirming that SnRK1.1 interacts with E2Fa *in vivo*. To gain additional insights into the regulatory mechanism involved in the SnRK1.1‐dependent E2Fa repression, we investigated SnRK1.1‐binding region(s) in E2Fa. The Y2H assay showed that the E2Fa^282–352^ fragment including MB domain, a potential heterodimerization and DNA bending motif (Mariconti *et al*., 2002), is responsible for the SnRK1.1 interaction (Fig. 4b).

We carried out kinase assay for *in vivo* confirmation of E2Fa phosphorylation by SnRK1.1. The immunocomplex SnRK1.1^WT^ did phosphorylate GST‐E2Fa but not GST‐E2Fc (Fig. 4c). SnRK1.1^IN^ could not phosphorylate GST‐E2Fa, confirming that SnRK1.1 interacts with and phosphorylates E2Fa.

Consistent with the finding that SnRK1.1 interacts with the MB domain, subsequent bioinformatics analysis identified T314, T315, and T339 as the potential SnRK1.1‐dependent phosphorylation sites (Fig. S6a), based on the conserved SnRK1.1‐binding‐phosphorylation motif (Huang & Huber, 2001; Halford *et al*., 2003; Vlad *et al*., 2008; Nukarinen *et al*., 2016). To precisely map the phosphorylation sites in the MB domain of E2Fa, we used liquid chromatography–tandem mass spectrometry (LC–MS/MS) to analyze in‐gel digested peptides of E2Fa^282–352^, followed by *in vitro* SnRK1.1 kinase assay. Phosphorylation of T315 was identified with high confidence (SpecEvalue of 1.00 × 10^−18^) with an observation of the x_16_*^2+^ ion (Fig. 4d) (Kelstrup *et al*., 2011), suggesting that T315 in the E2Fa MB domain serves as the major phosphorylation site of SnRK1.1 kinase.

To validate the SnRK1.1‐dependent E2Fa phosphorylation site (T315) identified by the LC–MS/MS, we performed *in vitro* SnRK1.1 kinase assay with GST‐tagged WT MB (E2Fa^282–352^) (GST‐E2FaMB) and a variant GST‐E2FaMB^T315A^ harboring an alanine substitution (T315A) that blocks phosphorylation. We also included GST‐E2FaMB^T314A^ and E2FaMB^T314AT315A^ in the assay since this amino acid next to T315 was also predicted as a phosphorylation site in our bioinformatics analysis. GST‐E2FaMB was phosphorylated, and GST‐E2FaMB^T314A^ was phosphorylated weakly by SnRK1.1 (Fig. 4e). However, SnRK1.1 did not phosphorylate GST‐E2FaMB^T315A^ or GST‐E2FaMB^T314AT315A^. These results indicate that T315 serves the major SnRK1.1 phosphorylation site of the MB domain in E2Fa and T314 may also be involved. E2Fa T314 and T315 are conserved in E2Fb, but not in E2Fc (Fig. S6b), further supporting that E2Fa and E2Fb, but not E2Fc, are key cell cycle transcription activators under the regulation of SnRK1.1‐mediated phosphorylation.

Since SnRK1.1 phosphorylated E2FaMB^T314A^ relatively weakly (Fig. 4e) and MS analysis identified only T315 as a phosphorylation site (Fig. 4d), we hypothesized that T314 and T315 might be phosphorylated in a sequential manner. Furthermore, since our bioinformatics analysis identified not only T314 and T315 but also T339 as the potential SnRK1.1‐dependent phosphorylation site (Fig. S6a), their phosphorylation could perhaps lead to subsequent phosphorylation of S338 or T339. To test this hypothesis, we examined whether E2FaMB^T315D^, which mimics phosphorylation at T315, could promote subsequent phosphorylation at other sites. SnRK1.1 phosphorylated E2FaMB^T315D^ to a certain level, but not E2FaMB^T314AT315D^ (Fig. 4f). By contrast, SnRK1.1 did not phosphorylate E2FaMB^T314DT315D^, E2FaMB^T314DT315DS338A^, or E2FaMB^T314DT315DT339A^. These results indicate that SnRK1.1 phosphorylates T315 and T314 in the E2Fa MB domain, but their phosphorylation does not result in subsequent phosphorylation of S338 and T339.

### SnRK1.1 negatively modulates E2Fa and E2Fb protein stability through 26S proteasome‐mediated protein degradation

**Fig. 5:**
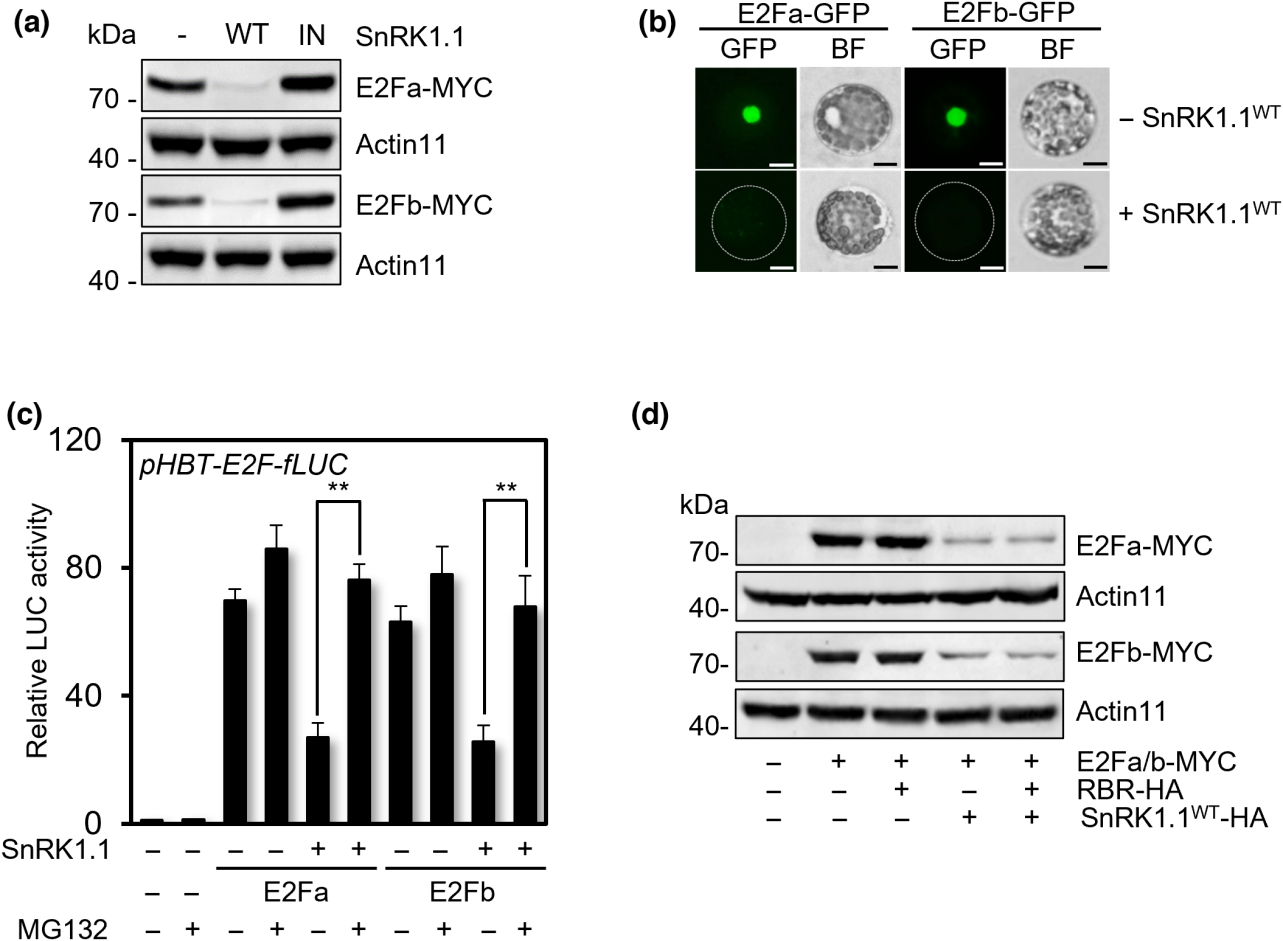
SnRK1.1 degrades E2Fa/b proteins. (a) Protein blot analysis of E2Fa and E2Fb with a combination of SnRK1.1^WT^ and SnRK1.1^IN^ co‐expression using anti‐MYC antibody. C‐terminal MYC‐conjugated E2Fa and E2Fb were co‐expressed in the protoplasts with SnRK1.1^WT^ or SnRK1.1^IN^ for protein blot analysis using anti‐MYC antibody. Actin11 served as a control. (b) Cellular images of E2Fa‐GFP and E2Fb‐GFP with and without SnRK1.1^WT^ co‐expression. C‐terminal GFP‐conjugated E2Fa and E2Fb wereexpressed in the protoplasts with or without SnRK1.1. Bar, 10 μm. (c) Measurements of E2Fa/b translation reporter activity in the protoplasts with and without SnRK1.1^WT^ co‐expression in the presence and absence of the proteasome inhibitor MG132. C‐terminal *fLUC*‐conjugated *E2Fa/b* were expressed in the protoplasts under *HBT* promoter with or without SnRK1.1 and MG132. *pUBQ10‐rLUC* served as a control. Values are mean ± SD. Asterisks indicate values statistically different from control (*t*‐test; **, *P* < 0.01). (d) Protein blot analysis of E2Fa‐MYC and E2Fb‐MYC with or without SnRK1.1^WT^ and RBR‐HA co‐expression using anti‐MYC antibody. Actin11 served as a control.

In light of the findings that E2Fa activity is post‐translationally regulated (Figs 2, 3) and SnRK1.1 phosphorylates E2Fa (Fig. 4), we analyzed E2Fa and E2Fb protein levels with co‐expression of either SnRK1.1^WT^ or SnRK1.1^IN^. Both E2Fa and E2Fb protein levels were reduced in the *Arabidopsis* protoplasts co‐expressing SnRK1.1^WT^ but not by SnRK1.1^IN^ co‐expression (Fig. 5a). Consistent with the protein blot analysis results, the fluorescence signal of GFP‐tagged E2Fa and E2Fb in the nucleus was also compromised by SnRK1.1^WT^ co‐expression (Fig. 5b). To further confirm these findings, we used protein translation reporters constructed by translational fusion of either *E2Fa* or *E2Fb* cDNA to *fLUC* reporter gene under the control of *35SC4PPDK* promoter. The fLUC activity of the protein translation reporters reflects the protein accumulation levels of the fusion proteins. First, E2Fa‐fLUC and E2Fb‐fLUC activities were significantly reduced by SnRK1.1 co‐expression, while E2Fa‐fLUC and E2Fb‐fLUC activities were maintained in the presence of the proteasome inhibitor MG132 (Fig. 5c), suggesting that SnRK1.1 negatively modulates E2Fa and E2Fb protein stability through 26S proteasome‐mediated protein degradation.

In cell cycle regulation, RETINOBLASTOMA‐RELATED‐PROTEIN (RBR) negatively regulates E2F‐dependent activation of S‐phase genes (Harashima & Sugimoto, 2016). We asked whether SnRK1.1 activity affects RBR‐dependent regulation of E2Fa/b function. Fig. 5d shows that SnRK1.1‐mediated suppression of E2Fa/b activity was not altered by RBR co‐expression, indicating that SnRK1.1 and RBR may repress E2Fa/b in different manners. Moreover, RBR did not affect E2Fa/b protein accumulations, while SnRK1.1 reduced E2Fa/b protein levels regardless of RBR co‐expression (Fig. 5d). These results suggest that SnRK1.1 suppresses E2Fa/b functions through their protein degradation, which is distinct from the RBR‐dependent negative transcriptional regulation of E2F during cell cycle progression.

### SnRK1.1‐mediated E2Fa degradation restricts primary root growth

**Fig. 6:**
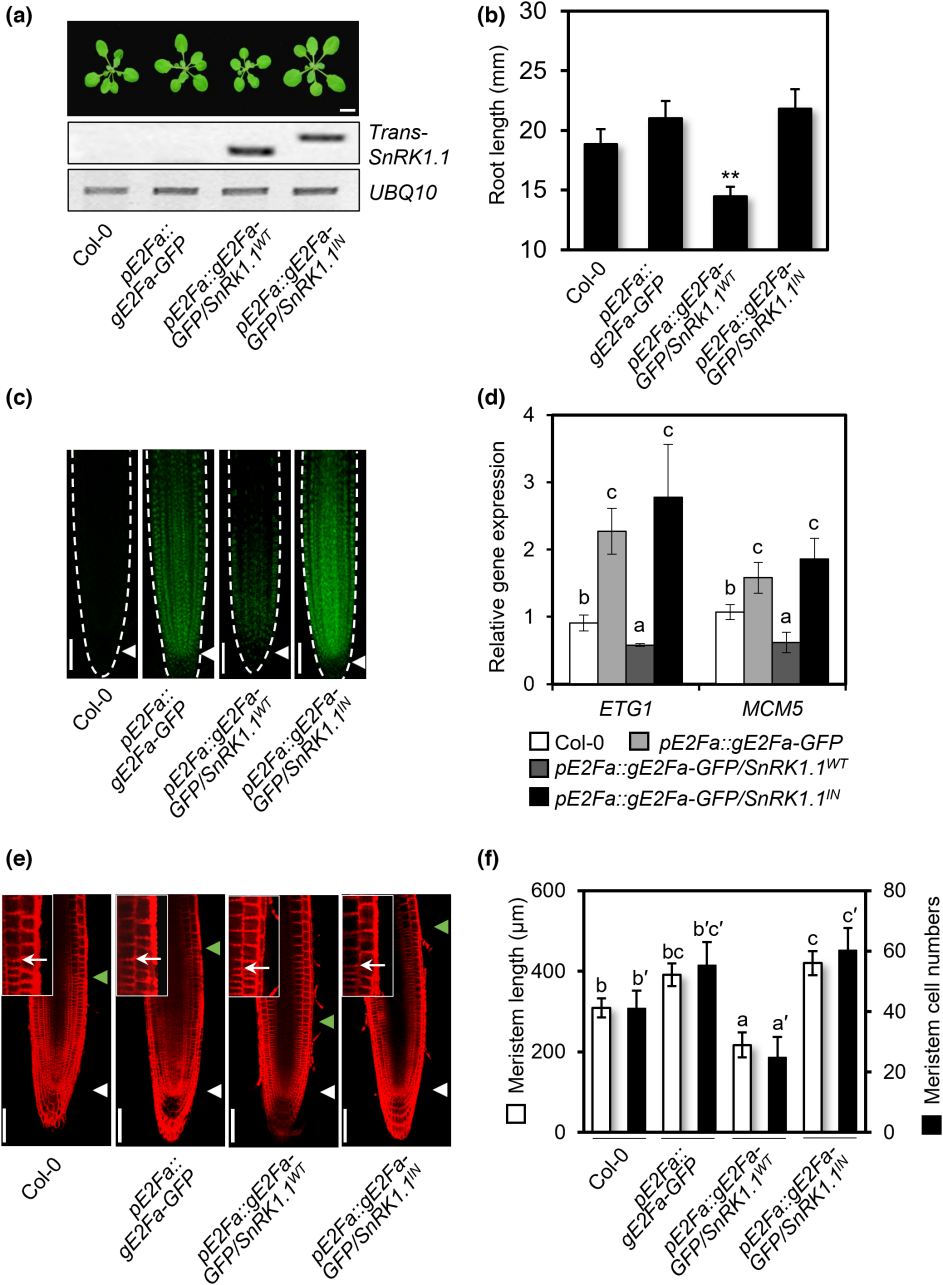
SnRK1.1 kinase activity leads to *Arabidopsis* growth restriction through E2Fa repression. (a) Growth phenotypes of *Arabidopsis* rosettes of 21‐d‐old designated genotypes. dCAPs analysis of *SnRK1.1* ^*WT*^ and *SnRK1.1* ^*IN*^. *UBQ10* was served as a control. Bar, 20 mm. (b) Quantification analysis of primary root length of designated genotypes. The plants were grown in ½MS liquid medium containing 0.5% sucrose for 5 d. Values are mean ± SD. Asterisks indicate statistical differences according to *t*‐test (**, *P* < 0.01). (c) Cellular images of E2Fa‐GFP in root apical meristems. The Col‐0, *pE2Fa::gE2Fa‐GFP*, *pE2Fa::gE2Fa‐GFP*/*SnRK1.1* ^*WT*^, and *pE2Fa::gE2Fa‐GFP*/*SnRK1.1* ^*I*^ plants were grown in ½MS liquid medium containing 0.5% sucrose for 5 d. The image was taken with fluorescence microscope. Arrowheads: quiescent center. Bar, 100 μm. (d) Expression analysis of cell cycle‐related genes in Col‐0, *pE2Fa::gE2Fa‐GFP*, *pE2Fa::gE2Fa‐GFP*/*SnRK1.1* ^*WT*^, and *pE2Fa::gE2Fa‐GFP*/*SnRK1.1* ^*IN*^. The plants were grown in ½MS liquid medium containing 0.5% sucrose for 5 d. Total RNA was extracted from the plants and carried out cDNA synthesis with the RNA. qPCR was carried out with gene‐specific primers. *EIF4a* was served as a control. Values are mean ± SD. Different letters indicate statistical differences according to ANOVA (*P* < 0.05). (e, f) Cellular images (e) and quantitative analysis (f) of root apical meristems of 5‐d‐old Col‐0, *pE2Fa::gE2Fa‐GFP*, *pE2Fa::gE2Fa‐GFP*/*SnRK1.1* ^*WT*^, and *pE2Fa::gE2Fa‐GFP*/*SnRK1.1* ^*IN*^. The plants were grown in ½MS liquid medium containing 0.5% sucrose for 5 d and stained with FM4‐64 solution. White arrowheads, quiescent center; green arrowheads, cell division–expansion transition zone. Enlarged cellular images near green arrows were inserted at top left corner. Bar, 100 μm. Values are mean ± SD. Different letters indicate statistical differences according to ANOVA (*P* < 0.05). All experiments were repeated at least three times with similar results.

To genetically conclude that SnRK1.1‐mediated phosphorylation‐dependent degradation of E2Fa leads to organ growth restriction, *pE2Fa*::*gE2Fa‐GFP* transgenic plants were crossed with *SnRK1.1* ^*WT*^ or *SnRK1.1* ^*IN*^ transgenic plants to generate *pE2Fa*::*gE2Fa‐GFP*/*SnRK1.1* ^*WT*^ and *pE2Fa*::*gE2Fa‐GFP*/*SnRK1.1* ^*IN*^ transgenic plants. *SnRK1.1* ^*WT*^ and *SnRK1.1* ^*IN*^ transgenes were confirmed in the F_3_ double homozygous transgenic lines using dCAPS analysis (Fig. 6a), and the HA‐epitope‐tagged SnRK1.1^WT^ and SnRK1.1^IN^ were clearly detected in the transgenic lines using the anti‐HA antibody (Fig. S7). SnRK1.1^WT^‐HA, but not SnRK1.1^IN^‐HA, was further detected using the anti‐pT172‐AMPKα antibody, indicating that only SnRK1.1^WT^ was phosphorylated at its T‐loop in the transgenic lines. Under normal growth condition, primary root length was shorter in the *pE2Fa*::*gE2Fa*‐*GFP*/*SnRK1.1* ^*WT*^ plants than that of Col‐0, *pE2Fa*::*gE2Fa*‐*GFP*, or *pE2Fa*::*gE2Fa*‐*GFP*/*SnRK1.1* ^*IN*^ (Fig. 6b). Likewise, the fluorescence signal of E2Fa‐GFP was not detected in the primary roots of the *pE2Fa*::*gE2Fa*‐*GFP*/*SnRK1.1* ^*WT*^ seedlings but was detected in the *pE2Fa*::*gE2Fa*‐*GFP*/*SnRK1.1* ^*IN*^ plants (Fig. 6c). Moreover, the expression of E2Fa target genes *ETG1* and *MCM5* was reduced in the *pE2Fa*::*gE2Fa*‐*GFP*/*SnRK1.1* ^*WT*^ plants, compared with their expression in the *pE2Fa*::*gE2Fa*‐*GFP*/*SnRK1.1* ^*IN*^ plants (Fig. 6d). Furthermore, primary meristem size and number were decreased in the *pE2Fa*::*gE2Fa*‐*GFP*/*SnRK1.1* ^*WT*^ plants, compared with their expression in the *pE2Fa*::*gE2Fa*‐*GFP*/*SnRK1.1* ^*IN*^ plants (Fig. 6e,f). Taken together, these data confirm that SnRK1.1‐mediated phosphorylation‐dependent degradation of E2Fa leads to S‐phase repression and growth restriction of *Arabidopsis* primary roots.

### ES‐SnRK1.1 signaling‐dependent phosphorylation at E2Fa T314/T315 residues leads to E2Fa protein degradation

**Fig. 7:**
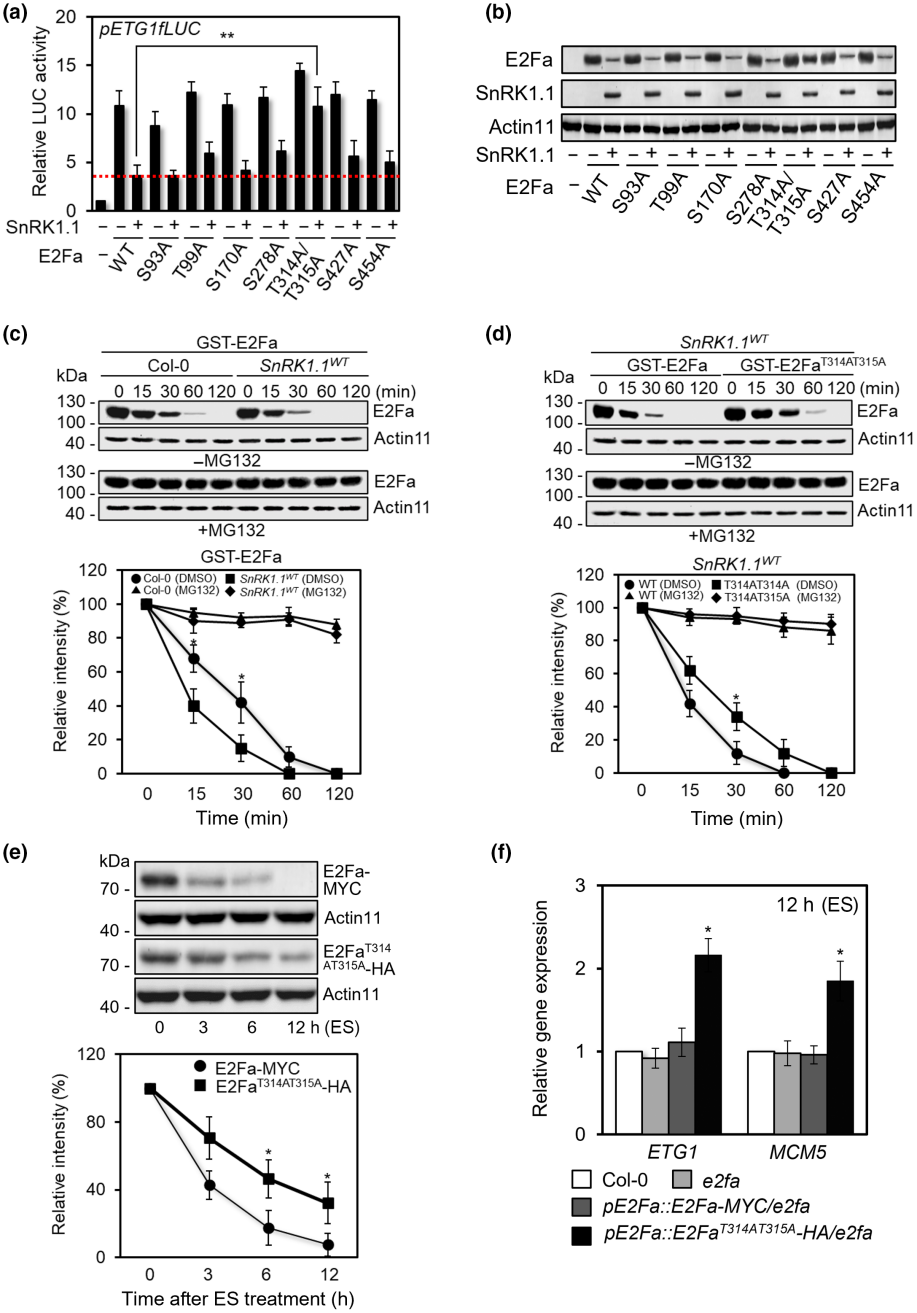
SnRK1.1‐dependent phosphorylation of E2Fa residues T314 and T315 leads to E2Fa protein degradation. (a) Promoter activities of *ETG1* with combination of E2Fa, E2Fa variants, and SnRK1.1 co‐expression in protoplasts. *pETG1‐fLUC* was transfected to protoplasts with designated effector combinations. *pUBQ10‐rLUC* served as a control. Values are mean ± SD. Asterisks indicate values statistically different from control (*t*‐test; **, *P* < 0.01). (b) Protein blot analysis for protein accumulation of E2Fa and E2Fa variants with or without SnRK1.1 co‐expression in protoplasts. Protein blot analysis was carried out using anti‐HA antibody. Actin11 served as a control. (c) Protein blot and quantitative analyses of cell‐free protein degradation assay of GST‐E2Fa with the protein extracts of Col‐0 or *SnRK1.1* ^*WT*^ transgenic plants in the presence and absence of MG132. Actin11 served as a control. Values are mean ± SD. (d) Protein blot and quantitative analyses of cell‐free protein degradation assay of GST‐E2Fa and GST‐E2Fa^T314AT315A^ with the protein extracts of *SnRK1.1* ^*WT*^ transgenic plants in the absence and presence of MG132. Actin 11 served as a control. Values are mean ± SD. Asterisk indicates values statistically different (*t*‐test; *, *P* < 0.05). (e) Protein blot and quantitative analyses of E2Fa in *pE2Fa::E2Fa‐MYC* or *pE2Fa::E2Fa* ^*T314AT315A*^ *‐HA* transgenic plants after energy stress (ES) treatment. Four‐day‐old seedlings were treated with ES for indicated times. Total protein was isolated from the plants and carried out protein blot analysis with anti‐MYC antibody. Actin11 served as a control. Values are mean ± SD. Asterisks indicate values statistically different (*t*‐test; *, *P* < 0.05). (f) Gene expression analysis of *ETG1* and *MCM5* in *pE2Fa::E2Fa‐MYC* or *pE2Fa::E2Fa* ^*T314AT315A*^ *‐HA‐*expressing plants after ES treatment. Four‐day‐old seedlings were treated with ES for 12 h and carried out RT‐qPCR. *EIF4a* served as a control. Values are mean ± SD. Asterisks indicate values statistically different from Col‐0 control (*t*‐test; *, *P* < 0.05). [Correction added after online publication 7 December 2022: labels in panels (b, e, f) have been updated.]

To further gain insights into the SnRK1.1‐mediated post‐translational regulation of E2Fa, we systematically examined protein stability and target gene regulation of E2Fa and E2Fa variants harboring Ala substitution at the additional putative phosphorable Ser and Thr residues identified in our bioinformatics analysis (Figs S6a, S8). To assess the transcriptional activity of E2Fa variants in the presence of SnRK1.1, we measured pETG1‐fLUC reporter activity in the protoplasts expressing E2Fa, E2Fa^S93A^, E2Fa^T99A^, E2Fa^S170A^, E2Fa^S278A^, E2Fa^T314AT315A^, E2Fa^S427A^, or E2Fa^S454A^ with or without SnRK1.1. When co‐expressed with SnRK1.1, E2Fa and E2Fa variant‐inducible pETG1‐fLUC activities were repressed as their protein accumulation was largely compromised by SnRK1.1 (Fig. 7a,b). However, E2Fa^T314AT315A^‐inducible pETG1‐fLUC activity was not altered by SnRK1.1 as the E2Fa^T314AT315A^ mutant is not phosphorylated by SnRK1.1 and, therefore, not subject to phosphorylation‐dependent protein degradation.

We then hypothesized that phosphorylation of T314 and T315 of E2Fa by SnRK1.1 leads to phosphorylation‐dependent degradation of the protein. To test this hypothesis, we carried out cell‐free protein turnover assay for GST‐E2Fa and GST‐E2Fa^T314AT315A^. Protein abundance was measured for GST‐E2Fa or GST‐E2Fa^T314AT315A^ incubated in the protein extracts isolated from WT Col‐0 or *SnRK1.1* ^*WT*^ transgenic plants. GST‐E2Fa decreased faster in the protein extracts of *SnRK1.1* ^*WT*^ transgenic plants than in the extracts of Col‐0 (Fig. 7c). On the contrary, E2Fa protein level maintained in the presence of protease inhibitor MG132, verifying 26S proteasome‐mediated degradation of E2Fa. As predicted, degradation of GST‐E2Fa^T314AT315A^ was slower than that of GST‐E2Fa in the protein extracts isolated from *SnRK1.1* ^*WT*^ transgenic plants (Fig. 7d).

To further confirm that E2Fa phosphorylation regulates the protein stability of E2Fa *in planta* during ES, we generated transgenic plants expressing *pE2Fa::E2Fa‐MYC* or *pE2Fa::E2Fa* ^*T314AT315A*^ *‐HA* in *e2fa* mutant background. Multiple homozygous single transgene insertion lines with high level of transgene expression were selected and used in the analysis (Fig. S9). Consistent with the results from *in vitro* assay of protein turnover, E2Fa^T314AT315A^–MYC protein accumulation was higher than that of E2Fa‐MYC in *e2fa* complemented transgenic plants under ES conditions (Fig. 7e). The expression of E2Fa target genes also was significantly higher in *pE2Fa::E2Fa* ^*T314AT315A*^ *‐HA/e2fa* than in *pE2Fa::E2Fa‐MYC/e2fa* under ES conditions (Fig. 7f). These data indicate that T314/T315 residues of E2Fa contribute to the protein degradation of E2Fa in plant under ES conditions.

### The two phosphorylation sites of E2Fa are critical for the ES‐induced root growth restriction

**Fig. 8:**
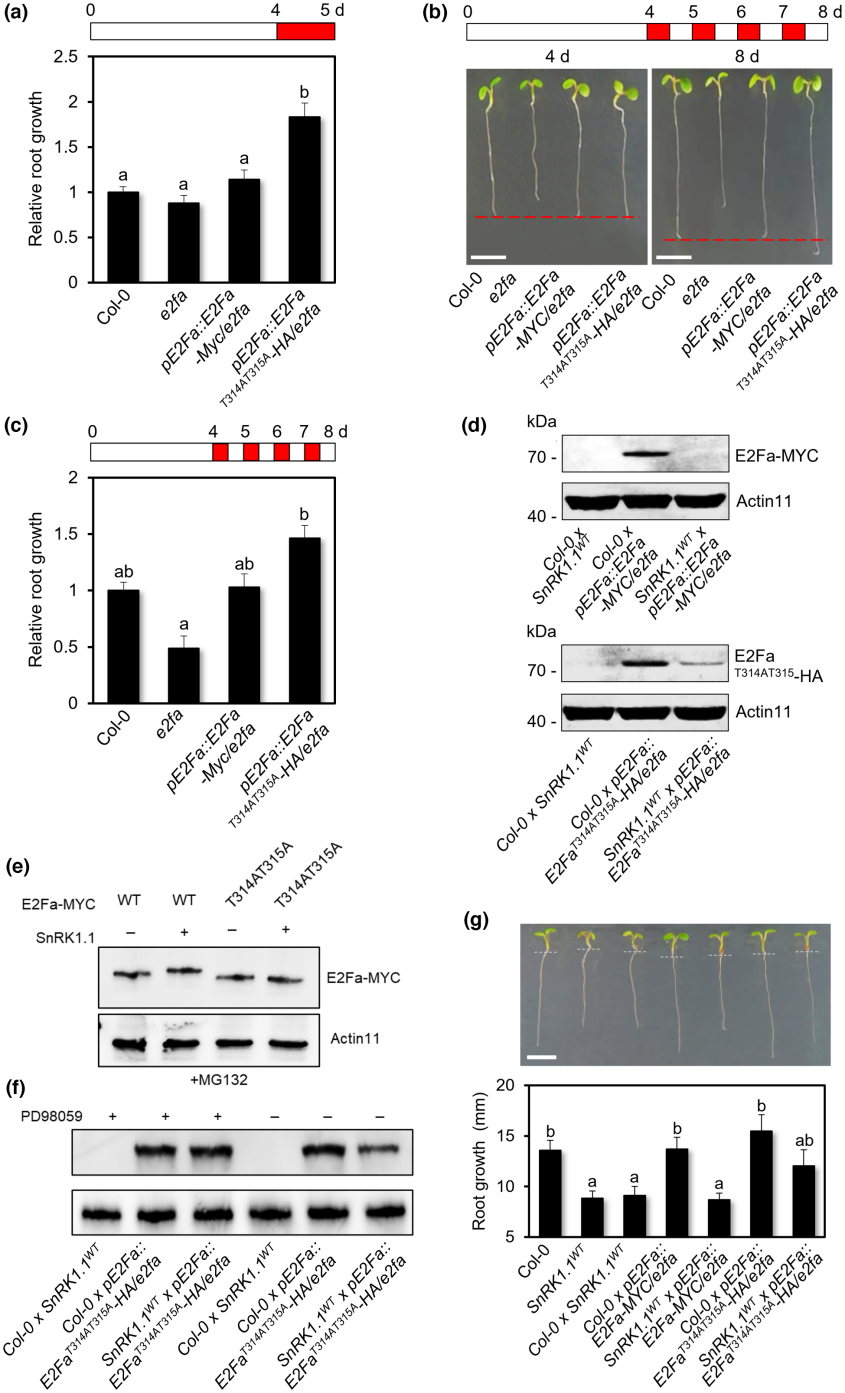
Root growth analysis and protein stability of F_1_ double heterozygous transgenic plants by crossing *E2Fa/e2fa* or *E2Fa* ^*T314AT315A*^ */e2fa* with *SnRK1.1*. (a) Quantitative analysis of primary root growth of Col‐0, *e2fa, pE2Fa::E2Fa‐MYC*, and *pE2Fa::E2Fa* ^*T314AT315A*^ *‐HA* after energy stress (ES) treatment. Four‐day‐old seedlings were treated with ES for 24 h and measured the root lengths. Values are mean ± SD. Different letters indicate statistical differences according to ANOVA (*P* < 0.05). (b, c) Image of primary root growth (b) and quantitative analysis of the root lengths (c) of Col‐0, *e2fa*, *pE2Fa::E2Fa‐MYC/e2fa*, or *pE2Fa::E2Fa* ^*T314AT315A*^ *‐HA/e2fa* after ES treatment. Four‐day‐old seedlings were treated with ES for indicated conditions. Values are mean ± SD. Different letters indicate statistical differences according to ANOVA (*P* < 0.05). All experiments were repeated at least three times with similar results. Red bars indicate the period that ES was applied. (d) Protein stability of E2Fa and E2Fa^T314AT315A^ in *SnRK1.1* ^*WT*^/*pE2Fa::E2Fa* ^*T314AT315A*^ *‐HA/e2fa* plants. Total protein was isolated from 5‐d‐old F_1_ double heterozygous transgenic seedlings and carried out protein blot analysis with anti‐MYC antibody. Actin11 served as a control. (e) Protein mobility shift assay of E2Fa and E2Fa and E2Fa^T314AT315A^. C‐terminal *MYC*‐conjugated *E2Fa* and *E2Fa* ^*T314AT315A*^ were expressed in protoplasts with or without *SnRK1.1* in the presence of MG132. Protein blot analysis was carried out with anti‐MYC antibody. (f) Protein stability of E2Fa^T314AT315A^ in SnRK1.1 crossed line with MPK inhibitor. Four‐day‐old seedlings were transferred to 100 μM PD98059 contained ½MS liquid media and additionally grown for 1 d. Total protein was isolated from the plants and carried out protein blot analysis with anti‐MYC antibody. (g) Primary root growth and quantitative analysis of F_1_ double‐heterozygous transgenic‐crossed *E2Fa/e2fa* or *E2Fa* ^*T314AT315A*^ */e2fa* with *SnRK1.1* ^*WT*^. Root growth was measured from 5‐d‐old seedlings grown on ½MS liquid media. Bar, 4 mm. Values are mean ± SD. Different letters indicate statistical differences according to ANOVA (*P* < 0.05). All experiments were repeated at least three times with similar results.

With ES treatment, primary roots of *pE2Fa::E2Fa* ^*T314AT315A*^ *‐HA/e2fa* plants grew longer than those of *pE2Fa::E2Fa‐MYC/e2fa* (Fig. 8a). To further confirm the ES‐induced root growth phenotype, we repeatedly applied 12‐h d^−1^ ES treatment for 4 d and measured the length of the primary roots. This iterative treatment showed the primary root of *pE2Fa::E2Fa* ^*T314AT315A*^ *‐HA/e2fa* plants grew apparently longer than did the *pE2Fa::E2Fa‐MYC/e2fa* under ES conditions (Fig. 8b,c).

To provide further evidence of protein phosphorylation‐dependent degradation of E2Fa by SnRK1.1, we generated several F_1_ double‐heterozygous transgenic lines by crossing *E2Fa* or *E2Fa* ^*T314AT315A*^/*e2fa* with Col‐0 or *SnRK1.1* ^*WT*^ transgenic plants (Fig. S10). Protein accumulation of E2Fa was compromised by SnRK1.1 in the double‐heterozygous transgenic lines under normal growth condition. Notably, protein accumulation of E2Fa^T314AT315A^ was higher than that of E2Fa^WT^, consistent with the finding that SnRK1.1 phosphorylates T314 and T315 of E2Fa to trigger subsequent degradation of the protein (Fig. 8d). Furthermore, phosphorylation‐dependent shift was not shown when SnRK1.1 is co‐expressed with E2Fa^T314AT315A^ (Fig. 8e).

Even though E2Fa^T314AT315A^ protein stability was higher than that of E2Fa in the presence of SnRK1.1, the protein stability was not robust in the crossed line (Fig. 8d). To find the reason, we repeated the experiment in the presence of mitogen‐activated protein kinase (MPK) inhibitor PD98059 since MPK is activated by SnRK1.1 (Cho *et al*., 2016) and E2Fa has six putative MPK‐binding motifs (ELM: http://elm.eu.org/). With the MPK inhibitor, E2Fa^T314AT315A^ was not degraded in the crossed line with *SnRK1.1* ^*WT*^ (Fig. 8f), suggesting that MPK‐mediated phosphorylation‐dependent degradation of E2Fa may be involved in the weak protein stability.

As expected, the primary roots of *SnRK1.1* ^*WT*^ plants, Col‐0 × *SnRK1.1* ^*WT*^ plants, and *SnRK1.1* ^*WT*^ × *pE2Fa*::*E2Fa*‐*MYC*/*e2fa* plants were shorter than those of WT Col‐0 (Fig. 8g), further confirming that SnRK1.1 promotes its protein degradation *via* phosphorylation of E2Fa at the T314/T315 residues, resulting in S‐phase suppression and root growth restriction.

## Discussion

Cell proliferation is a key determinant of organismal growth, progression of which is tightly controlled by cellular energy status in microbes, animals, and plants. Plants as a sessile organism coordinate cell proliferation and organismal growth with their surrounding environmental factors such as light and oxygen and nutrient availability. Here, we show that an evolutionarily conserved energy sensor protein kinase SnRK1.1 regulates cellular ES signaling imposed by low oxygen and darkness and suppresses key transcriptional activators E2Fa/b of cell cycle progression (Fig. S11). Canonically, CYC/CDK‐RBR axis is responsible for E2Fa/b modulation in eukaryotic cell division (Harashima *et al*., 2013). In a surprising twist, substantial evidence reported in this study suggests that SnRK1.1‐mediated phosphorylation and phosphorylation‐dependent degradation of E2Fa/b underlie plant growth restriction under ES conditions.

In *Arabidopsis*, plant glucose‐metabolic sensor TARGET OF RAPAMYCIN (TOR) kinase phosphorylates and activates E2Fa/b, which leads to the progression of S‐phase in the meristem cells. Activation of TOR‐E2F mechanism requires both glucose and light signals at the shoot apex, while glucose energy signal alone is sufficient at the root apex (Li *et al*., 2017). When the transcription factor E2Fa is phosphorylated by TOR kinase, it promotes G1‐S transition, resulting in *Arabidopsis* root growth promotion (Xiong *et al*., 2013). Previous studies showed that AMPK‐mediated REGULATORY‐ASSOCIATED PROTEIN OF TOR (RAPTOR) phosphorylation represses the TOR kinase activity and cell cycle under starvation conditions (Gwinn *et al*., 2008). In plants, SnRK1.1 interacts with RAPTOR1B *in vivo* and phosphorylated it *in vitro* (Nukarinen *et al*., 2016). Therefore, this mechanism was suggested to be conserved in plants despite differences in detail (Gonzalez *et al*., 2020). Furthermore, SnRK1.1 is translocated from nucleus to cytosol and reduces TOR activity in ABA response (Belda‐Palazón *et al*., 2022). However, crosstalk mechanisms of SnRK1 and TOR signaling in ES are elusive largely in plants. In the current study, we showed that SnRK1.1 directly phosphorylates the MB domain of E2Fa and subsequently degrades E2Fa protein. Since TOR‐E2Fa signaling is a key factor for root stem cell activity, SnRK1‐mediated E2Fa degradation results in the inhibition of TOR‐E2Fa signaling in an antagonistic manner. Taken together, the signaling protein kinases as fundamental regulatory modules of cell cycle dynamically integrate cellular energy status into the fine‐tuning of plant organ growth.

A mammalian SNF1 homolog AMPK serves as a critical cancer repressor to inhibit G1‐S transition and cell proliferation (Hardie & Alessi, 2013). Growth inhibition and/or arrest is commonly associated with *SnRK1*‐expressing transgenic plants as well (Tomé *et al*., 2014; Broeckx *et al*., 2016; Hulsmans *et al*., 2016; Muralidhara *et al*., 2021). Counterintuitively, SnRK1 has also been reported to phosphorylate KRP6 and promote cell cycle progression (Guerinier *et al*., 2013). Since diverse cellular processes including apoptosis and autophagy are most likely triggered by *KPR6* expression (Liang *et al*., 2007), SnRK1‐inducible growth in *KRP6*‐expressing transgenic plants needs to be further tested whether cell cycle progression is the major cause of plant growth restoration and whether SnRK1 could either promote or terminate cell proliferation in different cellular contexts.

Although differential regulation of SnRK1.1 and SnRK1.2 was reported in various stress conditions (Fragoso *et al*., 2009; Sánchez‐Villarreal *et al*., 2018), SnRK1.1 and SnRK1.2 are functionally conserved as an α‐subunit of SnRK1 (Baena‐Gonzalez *et al*., 2007). In our study, the protein levels and activities of both SnRK1s were increased by ES conditions. Furthermore, single‐knockout mutants (*snrk1.1* or *snrk1.2*) did not produce any obvious phenotype by ES conditions (Fig. S2), reflecting their functional redundancy. For this reason, we relied on the use of *SnRK1.1* ^*IN*^ conferring dominant‐negative effect on SnRK1s. In addition, interestingly, in the *SnRK1.1* ^*WT*^ and *SnRK1.1* ^*IN*^ overexpressing transgenics, the endogenous SnRK1.1 proteins were not detected (Fig. 1c). This phenomenon was also shown in two different SnRK1 overexpressing lines (Baena‐Gonzalez *et al*., 2007; Cho *et al*., 2016). Since SnRK1 is a central regulator of ES signaling, it is prudent to think that there is a negative feedback mechanism controlling their endogenous protein level or activity through promoter activity or mRNA nontranslational regions (Van der Krol *et al*., 1990; Betti *et al*., 2021). However, it is remained to be uncovered.

SnRK1 kinase activation/phosphorylation is well documented under various extracellular stress conditions (Baena‐Gonzalez *et al*., 2007; Fragoso *et al*., 2009; Crozet *et al*., 2014; Sheen, 2014; Cho *et al*., 2016; Belda‐Palazón *et al*., 2020; Gutierrez‐Beltran *et al*., 2021). The protein kinase activity, activation of which is likely linked to its phosphorylation, is clearly involved in *Arabidopsis* organ growth modulation under ES conditions, at least in the early seedling stage (4–10 d after germination). Further mechanistic understanding of protein complex of SnRK1.1 is required to evaluate whether SnRK1.1 phosphorylation is attributed to its kinase activation. In the current study, SnRK1.1 kinase activity involved in plant growth restriction has been genetically validated with hyposensitive growth responses of *SnRK1.1* ^*IN*^ transgenic plants. Protein turnover and organ growth analyses of F_1_ double‐heterozygous transgenic plants expressed with *SnRK1.1* ^*WT*^and *E2Fa* or *E2Fa* ^*T314AT315A*^ have further provided compelling evidence for SnRK1.1 kinase‐inducible phosphorylation‐coupled E2Fa degradation, resulting in plant growth restriction. Identification of the E3 ligase(s) involved in the ubiquitination of phosphorylated E2Fa will yield insights into the detailed mechanism for protein degradation.

Likewise, E2Fb protein stability and its transcriptional activity were negatively regulated by SnRK1.1 (Figs 3, 5). However, its spatial gene expression profile was not correlated with that of *SnRK1.1* in root (Fig. S4d). While both E2Fa and E2Fb are involved in the coordination of cell proliferation, it was suggested that E2Fa and E2Fb could have different functions based on their association with components of the evolutionary conserved multi‐subunit DP‐Rb‐E2F and MuvB complex (Kobayashi *et al*., 2015; Leviczky *et al*., 2019). Therefore, our finding that SnRK1.1 differentially regulates E2Fa and E2Fb may support the hypotheses of differential function of the two.

Phosphorylation‐dependent conformational change affects the transcriptional activity or binding affinity of transcription factors (Kedage *et al*., 2017; Mizoi *et al*., 2019). MB domain of E2Fa is important for interaction with transcriptional partners (Black *et al*., 2005). In the current study, we show that SnRK1.1 phosphorylated the MB domain of E2Fa (Fig. 4), which resulted in the degradation of E2Fa (Fig. 5). It is prudent to think that the SnRK1.1‐mediated phosphorylation of the MB domain of E2Fa might affect its binding affinity with the transcriptional partner(s). However, the phosphorylation did not affect the transcriptional activity of E2Fa, evidenced by the high level of LUC reporter gene activity with the co‐expression of SnRK1.1 in the presence of MG132 (Fig. 5c). This suggests that the SnRK1.1‐mediated phosphorylation may not have an impact on the binding affinity of E2Fa with its transcriptional partner protein(s), while it leads to proteasome‐dependent degradation of the protein.

In conclusion, our study has unraveled a novel and direct regulation of E2F transcription factors by SnRK1.1, which allows the plants to dynamically adjust their vegetative growth in response to cellular energy status, hence serving as a robust adaptation mechanism to cope with constantly fluctuating environmental conditions.

## Author contributions

SS conceptualized the study; SS and JHI performed the experiments and data analysis; SS, JHI, J‐HK and K‐HH interpret the data and wrote the manuscript; YL and S‐WL performed the LC‐MS/MS experiments and analyzed LC‐MS/MS data; K‐HH supervised the study. All authors read and approved this paper. SS and JHI contributed equally to this work.

## Supporting information


**Fig. S1** Strategy of energy stress (ES) treatment and ES responses.
**Fig. S2** Primary root growth under the energy stress condition.
**Fig. S3** Organ growth rate in Col‐0, *SnRK1.1* ^*WT*^, *SnRK1.1* ^*IN*^, *pE2Fa::gE2Fa‐GFP*, and *E2Fa_RNAi*.
**Fig. S4** Y2H and *in vitro* kinase assay for E2Fa/b and SnRK1.1.
**Fig. S5** Co‐immunoprecipitation (Co‐IP) of E2Fc with SnRK1.1.
**Fig. S6** Consensus sequences of SnRK1 in E2Fa.
**Fig. S7** Protein blot analysis of *pE2Fa::gE2Fa‐GFP* transgenic plants co‐expressed with *SnRK1.1* ^*WT*^ *or SnRK1.1* ^*IN*^ for molecular validation.
**Fig. S8** Schematic draw of E2Fa protein structure.
**Fig. S9** Molecular validation of *E2Fa‐MYC/e2fa* and *E2Fa* ^*T314AT315A*^ *‐HA/e2fa* transgenic plants.
**Fig. S10** Semiquantitative analysis of transgenes expression in F_1_ double heterozygous transgenic lines.
**Fig. S11** Working model of SnRK1.1‐dependent E2F degradation.
**Table S1** Primers used in this study.
Please note: Wiley is not responsible for the content or functionality of any Supporting Information supplied by the authors. Any queries (other than missing material) should be directed to the *New Phytologist* Central Office.

## Data Availability

The data that support the findings of this study are openly available from the corresponding author upon reasonable request.
