# Supplementary material for: SNF1‐related protein kinase 1 represses Arabidopsis growth through post‐translational modification of E2Fa in response to energy stress
Source: New Phytol. 2022 Dec 7;237(3):823–39. doi: 10.1111/nph.18597 (PMC10107498; doi:10.1111/nph.18597)
Supplement: Supplementary file 1 — Fig. S1 Strategy of energy stress (ES) treatment and ES responses. Fig. S2 Primary root growth under the energy stress condition. Fig. S3 Organ growth rate in Col‐0, SnRK1.1 WT , SnRK1.1 IN , pE2Fa::gE2Fa‐GFP, and E2Fa_RNAi. Fig. S4 Y2H and in vitro kinase assay for E2Fa/b and SnRK1.1. Fig. S5 Co‐immunoprecipitation (Co‐IP) of E2Fc with SnRK1.1. Fig. S6 Consensus sequences of SnRK1 in E2Fa. Fig. S7 Protein blot analysis of pE2Fa::gE2Fa‐GFP transgenic plants co‐expressed with SnRK1.1 WT or SnRK1.1 IN for molecular validation. Fig. S8 Schematic draw of E2Fa protein structure. Fig. S9 Molecular validation of E2Fa‐MYC/e2fa and E2Fa T314AT315A ‐HA/e2fa transgenic plants. Fig. S10 Semiquantitative analysis of transgenes expression in F1 double heterozygous transgenic lines. Fig. S11 Working model of SnRK1.1‐dependent E2F degradation. Table S1 Primers used in this study. Please note: Wiley is not responsible for the content or functionality of any Supporting Information supplied by the authors. Any queries (other than missing material) should be directed to the New Phytologist Central Office. [file NPH-237-823-s001.pdf]

[Correction added after online publication 7 December 2022: The Supporting Information file has been updated.]

New Phytologist Supporting Information

Article title: SNF1-related protein kinase 1 represses *Arabidopsis* growth through post-translational modification of E2Fa in response to energy stress

Authors: Seungmin Son, Jong Hee Im, Jae-Heung Ko, Kyung-Hwan Han

Article acceptance date: 08 October 2022

Figure S1: Strategy of energy stress treatment and energy stress responses.

Figure S2: Primary root growth under energy stress condition.

Figure S3: Organ growth rate in Col-0, *SnRK1.1*^{WT}, *SnRK1.1*^{IN}, *pE2Fa::gE2Fa-GFP*, and *E2Fa_RNAi*.

Figure S4: Y2H and *in vitro* kinase assay for E2Fa/b and SnRK1.1.

Figure S5: Co-immunoprecipitation of E2Fc with SnRK1.1.

Figure S6: Consensus sequences of SnRK1 in E2Fa.

Figure S7: Protein blot analysis of *pE2Fa::gE2Fa-GFP* transgenic plants co-expressed with *SnRK1.1*^{WT} *or SnRK1.1*^{IN} for molecular validation.

Figure S8: Schematic draw of E2Fa protein structure.

Figure S9: Molecular validation of *E2Fa-MYC/e2fa* and *E2Fa*^{T314AT315A}*-HA/e2fa* transgenic plants.

Figure S10: Semi-quantitative analysis of transgenes expression in F_1 double heterozygous transgenic lines.

Figure S11: A working model of SnRK1.1 dependent E2F degradation.

Table S1: The primers used in this study.

Supplementary Figures

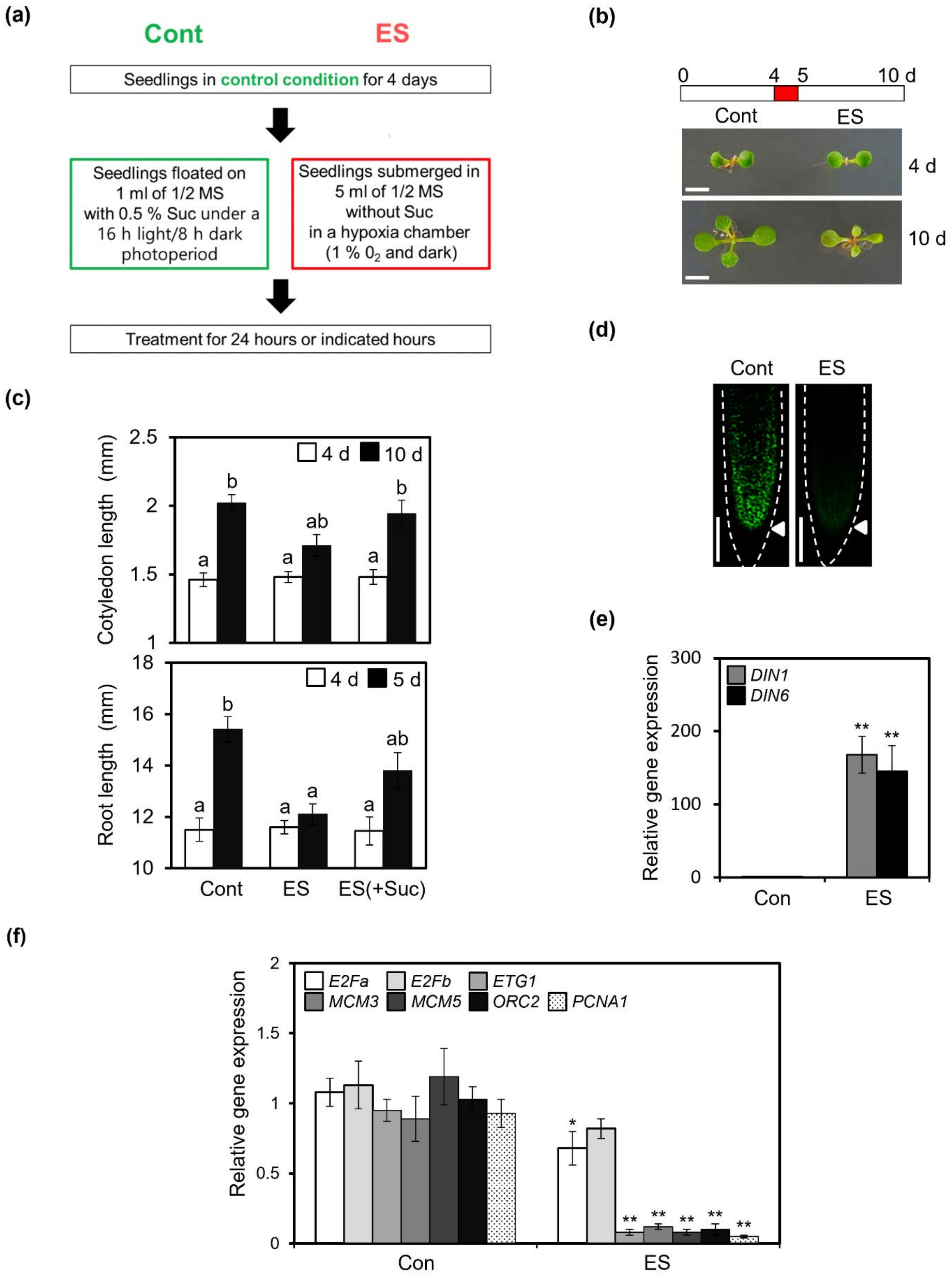

Fig. S1 Strategy of energy stress treatment and energy stress responses. (a) For energy stress (ES) treatment, *Arabidopsis* plants were grown in 6-well plates containing 1 ml of 1/2 MS liquid medium (0.5 % sucrose, pH 5.7 adjusted with KOH) for 4 days and transferred to a hypoxic chamber with 1 % O_2, < 0.1 % CO_2, and 98.9 % N_2 (Ruskinns $Invivo_{300}$) in submerged and dark conditions with or without carbon supplementation for indicated times. (b) Schematic diagram of ES treatment and growth phenotypes of Col-0 in Control and ES condition. White domain (control condition), plants were grown in 1 ml of 1/2 liquid MS medium (0.5% sucrose, pH 5.7 adjusted with KOH) at light for indicated days (d); Red domain (ES condition), plants were grown at a hypoxic chamber, submerged with half MS liquid medium (pH 5.7 adjusted with KOH) and dark conditions for indicated times. Scale bar: 2 mm. (c) Quantitative growth analysis of cotyledons and primary roots under ES with or without sucrose. Values are means ± SD. Different letters indicate statistical differences according to ANOVA ($P < 0.05$). (d) Cellular images of EdU staining in primary roots of Col-0 after ES, treatment on the 4-day-old seedling for 6 hr. Arrowheads: quiescent center. Scale bar: 100 μm. (e, f) Expression analysis of SnRK1.1. SnRK1.1-dependently inducible (e) and cell cycle-related genes (f) in Col-0 after ES treatment on the 4-day-old seedling for 24 hr using RT-qPCR. Total RNA was extracted from the seedings, and cDNA was synthesized with it. *EIF4a* was served as a control. Values are means ± SD. Asterisks indicate values statistically different from controls according to *t*-test (** $P < 0.01$, * $P < 0.05$).

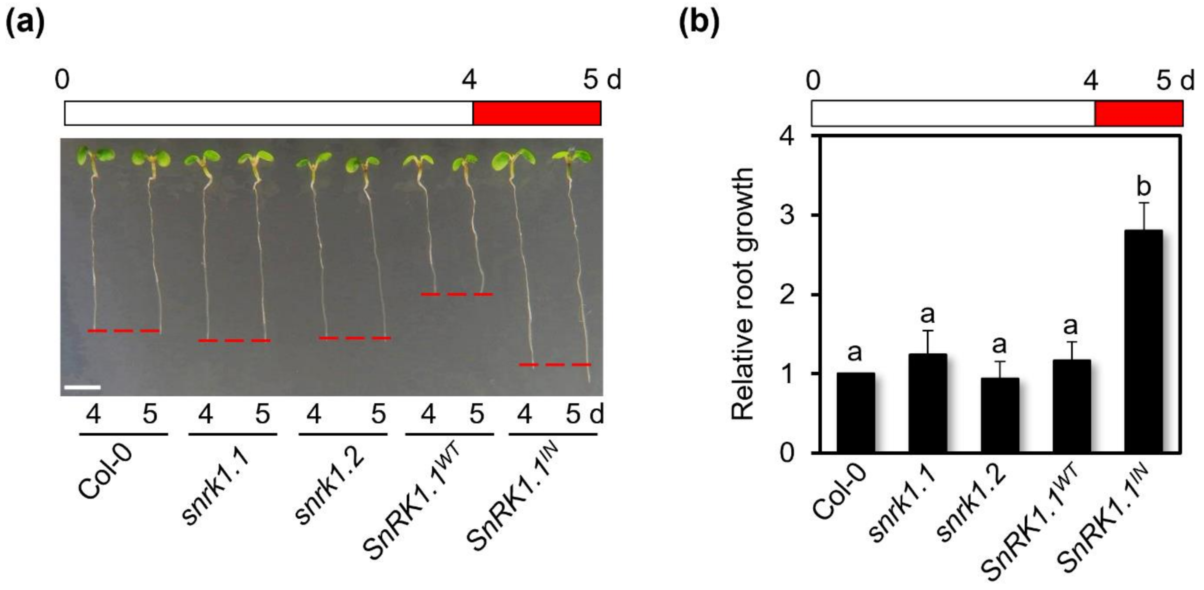

Fig. S2 Primary root growth under the energy stress condition. Primary root growth of Col-0, *snrk1.1*, *snrk1.2*, *SnRK1.1*^{WT}, and *SnRK1.1*^{IN} (a) and quantitative (b) analysis of 4-day-old Col-0, *snrk1.1*, *snrk1.2*, and transgenic plants-expressing *SnRK1.1*^{WT} or *SnRK1.1*^{IN} under ES condition for 24 hr. Scale bar: 4 mm. Values are means ± SD. Different letters indicate statistical differences according to ANOVA ($P < 0.05$).

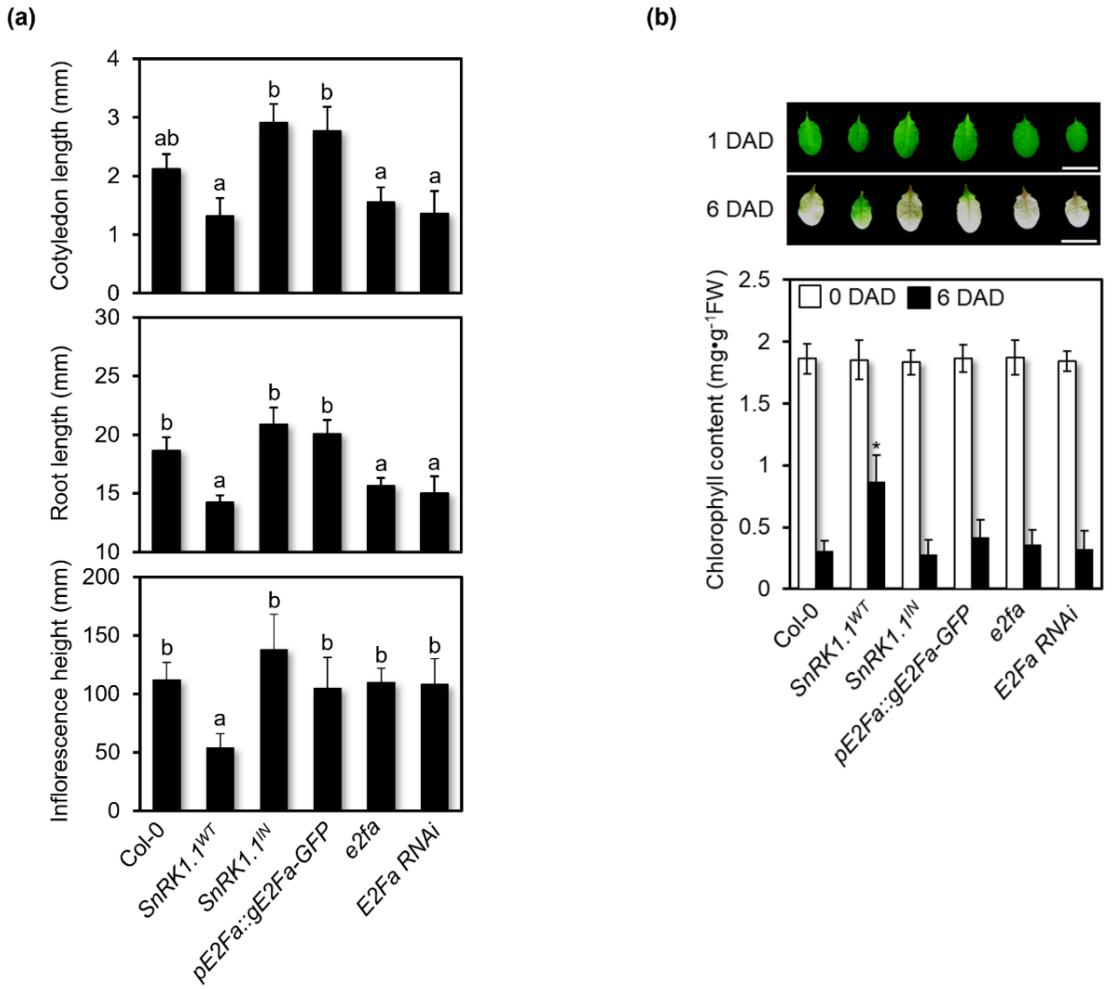

Fig. S3 Organ growth rate in Col-0, *SnRK1.1*^{WT}, *SnRK1.1*^{IN}, *pE2Fa::gE2Fa-GFP*, and *E2Fa_RNAi*. (a) Quantitative analysis of cotyledon growth, primary root length and inflorescence height in Col-0, transgenic plants-expressing designated gene. Cotyledon and root length were measured from Five-day old seedlings grown 0.5% sucrose contained 1/2 MS liquid medium, and inflorescence was measured from 25-day-old plants grown on soil. Scale bar: 2 mm. Values are means ± SD. Different letters indicate statistical differences according to ANOVA ($P < 0.05$). (b) Dark-induced senescence of Col-0, *SnRK1.1*^{WT}, *SnRK1.1*^{IN}, *pE2Fa::gE2Fa-GFP*, and *E2Fa_RNAi*. Leaves detached from 25-day-old plants left on wetted filter paper for 6 days in dark, and then the phenotype and chlorophyll contents were analyzed. Chlorophyll contents were measured as previously described (Porra *et al.*, 1989). DAD, day after darkness. Scale bar: 20 mm. Values are means ± SD. Asterisks indicate values statistically different from controls (*t*-test, * $P < 0.05$).

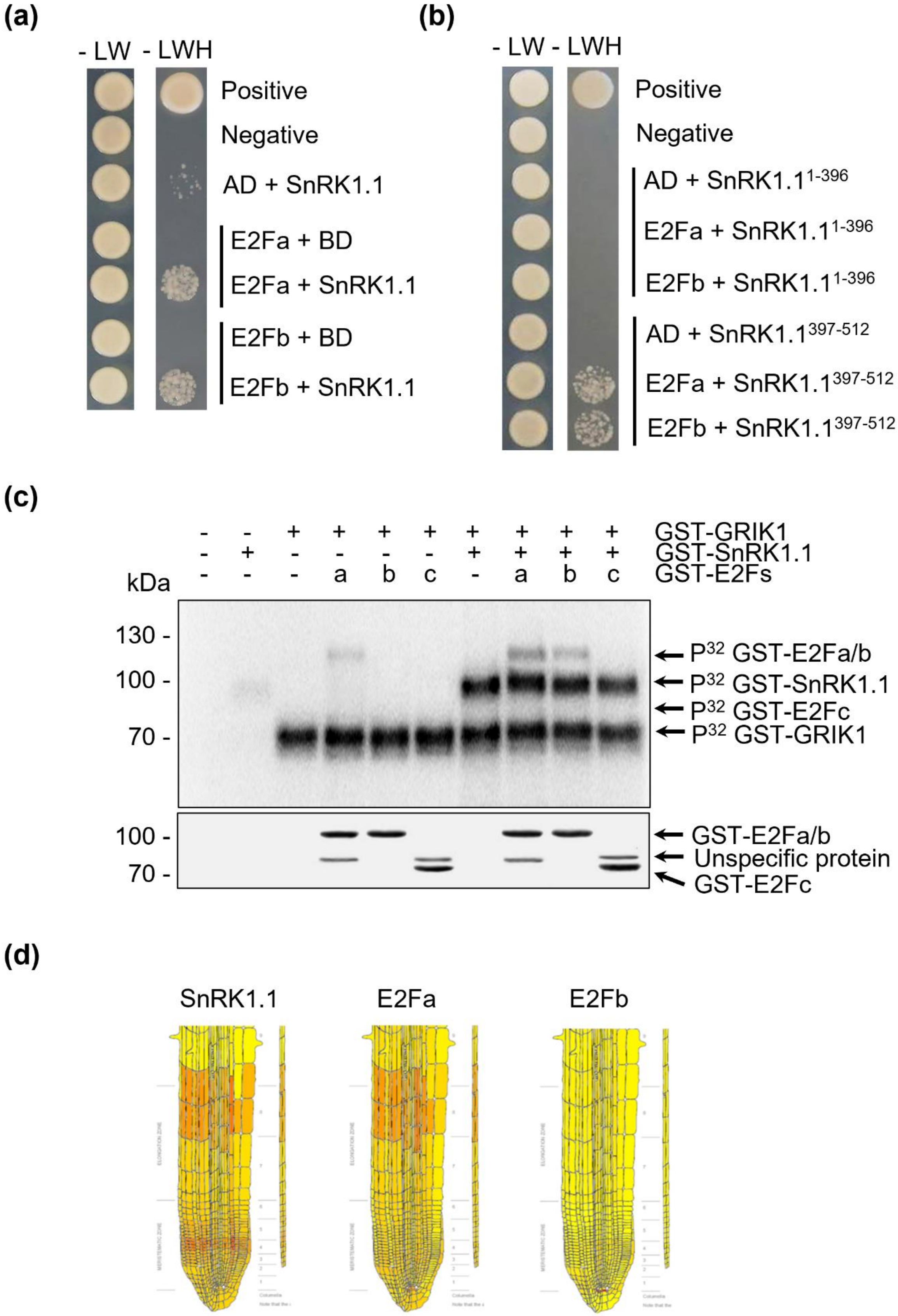
(a)
- LW
- LWH
Positive
Negative
AD + SnRK1.1
E2Fa + BD
E2Fa + SnRK1.1
E2Fb + BD
E2Fb + SnRK1.1
(b)
- LW
- LWH
Positive
Negative
AD + SnRK1.1 1-396
E2Fa + SnRK1.1 1-396
E2Fb + SnRK1.1 1-396
AD + SnRK1.1 397-512
E2Fa + SnRK1.1 397-512
E2Fb + SnRK1.1 397-512
(c)
GST-GRIK1
GST-SnRK1.1
GST-E2Fs
kDa
130 -
100 -
70 -
P32 GST-E2Fa/b
P32 GST-SnRK1.1
P32 GST-E2Fc
P32 GST-GRIK1
100 -
70 -
GST-E2Fa/b
Unspecific protein
GST-E2Fc
(d)
SnRK1.1
E2Fa
E2Fb

Fig. S4 Y2H and *in vitro* kinase assay for E2Fa/b and SnRK1.1. (a, b) Binary interaction assay between SnRK1.1 and E2Fa/b (a) and SnRK1.1 fragment and E2Fa/b (b) in a yeast two-hybrid system (Y2H). Yeast cells were grown in nutrient selection media containing 0.5 mM 3-amino-1,2,4-triazole (3-AT). (c) *In vitro* kinase assay of SnRK1.1 was carried out with E2Fa, E2Fb and E2Fc as substrates. Recombinant GST-tagged E2Fa, E2Fb, E2Fc, SnRK1.1 and GRIK1 were expressed and purified from *E. coli*. Kinase reactions were performed with P^{32}-[γ]-ATP for 30 min and then the reaction products were separated with SDS-PAGE. P^{32}-labeled proteins were visualized by autoradiography. (d) Expression of *SnRK1.1*, *E2Fa* and *E2Fb* in roots (*Arabidopsis* eFP Browser; http://bar.utoronto.ca). All experiments were repeated at least three times with similar results.

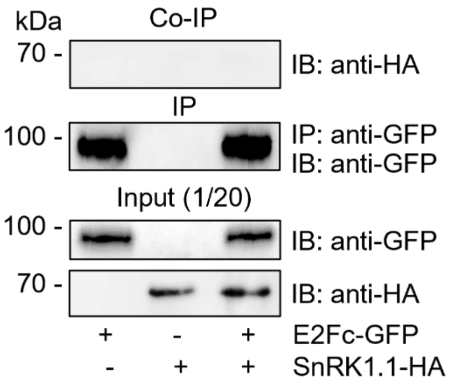

Fig. S5 Co-immunoprecipitation of E2Fc with SnRK1.1. C-terminal GFP conjugated E2Fc was expressed in protoplasts contained MG132 with or without SnRK1.1. After co-expression, IP was carried out with anti-GFP antibody.

(a)

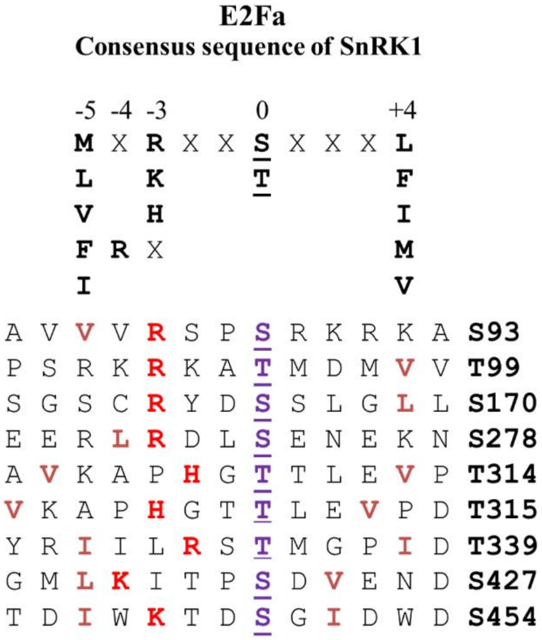

(b)

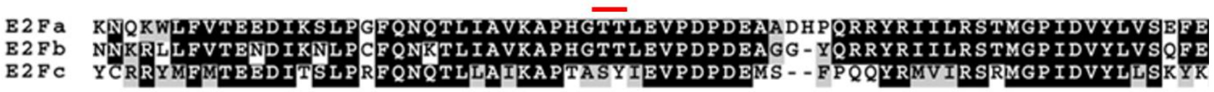

Fig. S6 Consensus sequences of SnRK1 in E2Fa. (a) Consensus sequences for SnRK1-binding and phosphorylation in E2Fa. (b) Amino acid sequence alignments around MB domain of E2Fa, E2Fb, and E2Fc. Red line indicates T314 and T315 of E2Fa.

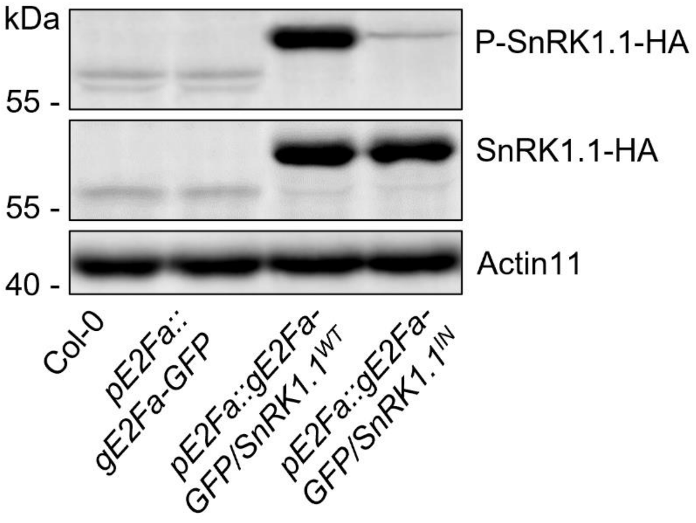

Fig. S7 Protein blot analysis of *pE2Fa::gE2Fa-GFP* transgenic plants co-expressed with *SnRK1.1*^{WT} *or SnRK1.1*^{IN} for molecular validation. Protein blot analysis of total SnRK1.1 and phosphorylated SnRK1.1 (P-SnRK1.1) of *pE2Fa::gE2Fa-GFP/ SnRK1.1*^{WT} and *pE2Fa::gE2Fa-GFP/ SnRK1.1*^{IN} transgenic lines. Actin11 served as a control. Experiment was repeated at least three times with similar results.

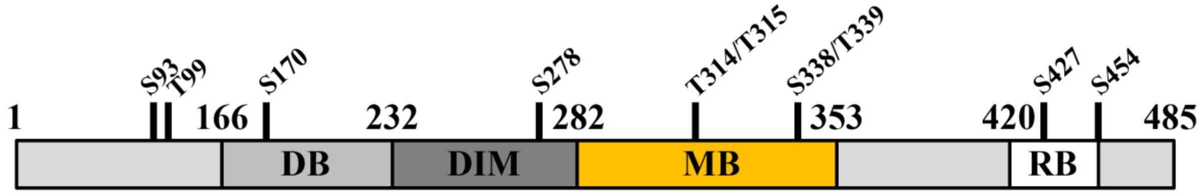

Fig. S8 Schematic draw of E2Fa protein structure. E2Fa has 10 putative SnRK1.1 phosphorylation sites, followed by bioinformatic analysis in Fig. S5. DB: DNA binding; DIM: dimerization; MB: marked box: RB: Rb binding domain.

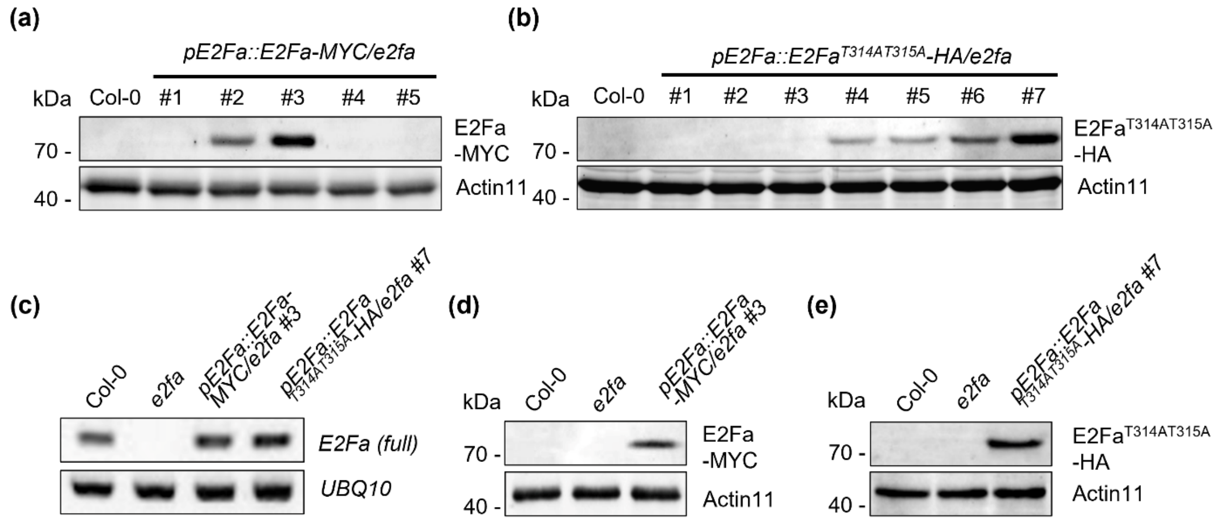

Fig. S9 Molecular validation of *E2Fa-MYC/e2fa* and *E2Fa*^{T314AT315A}*-HA/e2fa* transgenic plants. (a, b) Protein blot analysis of E2Fa and E2Fa^{T314AT315A} in multiple *pE2Fa::E2Fa-MYC/e2fa* lines (a) and *E2Fa*^{T314AT315A}*-HA/e2fa* lines (b). Ectopic protein expression of E2Fa in *E2Fa-MYC/e2f* lines was detected by anti-MYC antibody and E2Fa^{T314AT315A} in *E2Fa*^{T314AT315A}*-HA/e2fa* lines was detected by anti-HA antibody. Actin11 was served as a protein loading control. (c) Semi-quantitative analysis of *E2Fa* expression in *E2Fa-MYC/e2fa #3* and *E2Fa*^{T314AT315A}*-HA/e2fa #7* transgenic plants. *UBQ10* was served as a control. (d,e) Protein blot analysis of E2Fa-MYC (d) and E2Fa^{T314AT315A}-HA (e) in transgenic lines. Protein blot analysis was carried out with tag-specific antibodies. Actin11 served as a protein loading control. All experiments were repeated at least three times with similar results.

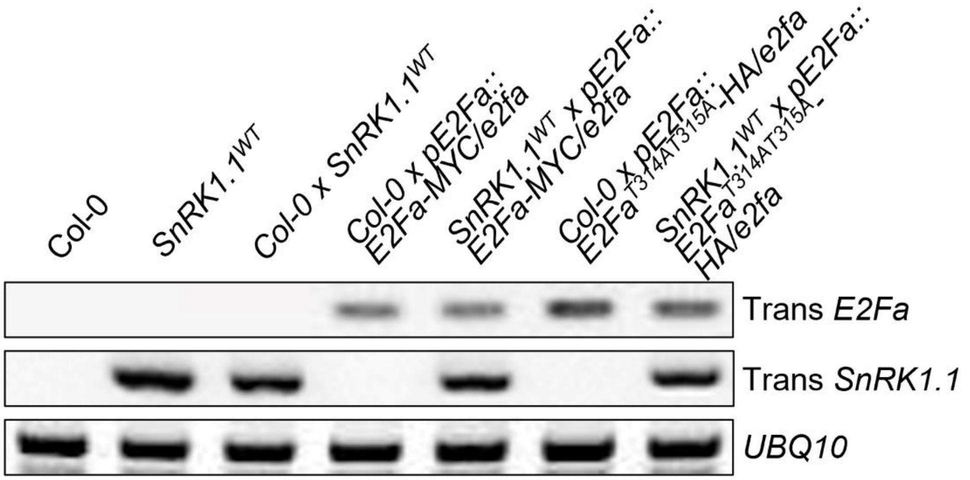

Fig. S10 Semi-quantitative analysis of transgenes expression in F_1 double heterozygous transgenic lines. F_1 double heterozygous transgenic lines were generated by crossing *E2Fa* or *E2Fa*^{T314AT315A}-expressing transgenic *e2fa* with Col-0 or *SnRK1.1*^{WT}-expressing transgenic Col-0 plants. *E2Fa* or *SnRK1.1* and *NOS-T* were respectively used as forward and reverse primers for transgene amplification. *UBQ10* served as a control.

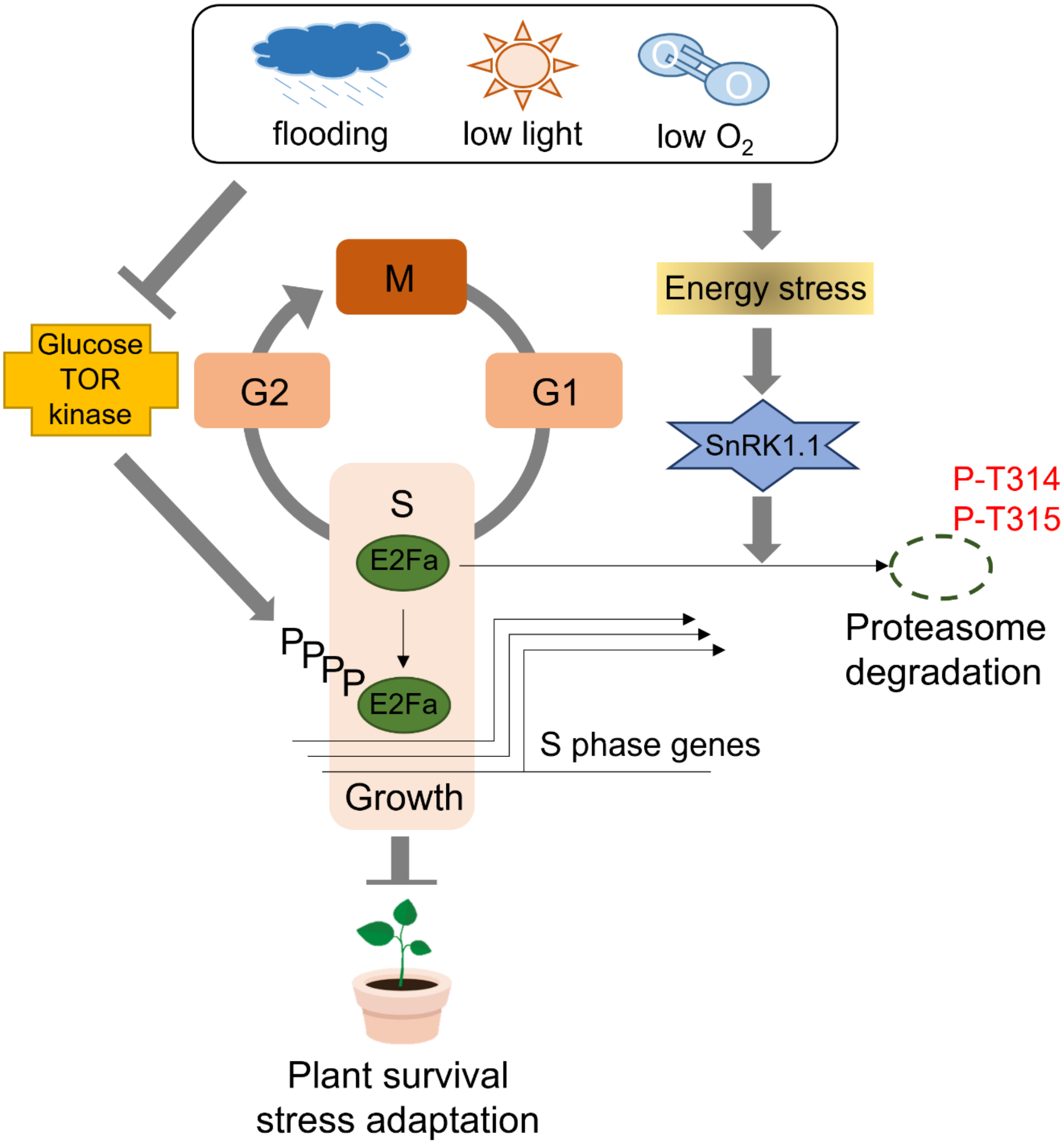

Fig. S11 A working model of SnRK1.1 dependent E2F degradation. Energy stress activated SnRK1.1 directly interacts with and phosphorylates the transcription factor E2Fa. SnRK1.1-dependent phosphorylation leads to E2Fa proteasome-dependent protein degradation and G1-S phase transition repression. As a result, SnRK1.1 restricts cell proliferation and vegetative organ growth for plants to survive in energy deprivation environments.

Supplementary Table

Table S1: The primers used in this study.

	Forward (5' → 3')	Reverse (5' → 3')	Purpose
E2Fa promoter	CCCAAGCTTAATTGGTTCTAGAGCTTTTAGTCC	CGGGATCCGGATCGTAGTGAAGCGAGAG	Cloning
ETG1 promoter	CGGGATCCAGCGAGCTGAGGGATTTGC	CATGCCATGGTCAAAACCAACCTTTGATTTCAC	
MCM5 promoter	CGGGATCCACTCTCTCTCTCTCTTTGAGG	CATGCCATGGTGCTTCGTCTCCGTTACGAA	
AKIN10	CGGGATCCATGGATGGATCAGGCACAG	GAAGGCCTGAGGACTCGGAGCTGAGCAAG	
AKIN10 1-396	CGGGATCCATGGATGGATCAGGCACAG	GAAGGCCTCTGAAGTCCAAGAGCCCAT	
AKIN10 397-512	CGGGATCCATGTCTCGGGCTCATCC	GAAGGCCTGAGGACTCGGAGCTGAGCAAG	
GRIK1	CGGGATCCATGTTTTGTGATAGTTTTGC	GAAGGCCTGCTATGGTTTTGATCTTCTTC	
E2Fa	CGGGATCCATGTCCGGTGTCGTACG	GAAGGCCTTCTCGGGGTTGAGTCAACA	
E2Fb	CGGGATCCATGTCTGAAGAAGTACCTCA	GAAGGCCTGCTACCTGTAGGTGATCTCGTAG	
E2Fc	CGGGATCCATGGCCGCGACATCAAACT	GAAGGCCTGCTGTTGAAGTTGCTCCATAA	
E2Fa 1-281	CGGGATCCATGTCCGGTGTCGTACG	GAAGGCCTTTCATTTTCGCTCAGGTC	
E2Fa 282-313	CGGGATCCATGAAGAATCAGAAATGGCTT	GAAGGCCTGCCATGAGGAGCTTTGA	
E2Fa 282-352	CGGGATCCATGAAGAATCAGAAATGGCTT	GAAGGCCTTTCAAATTCGCTGACGAGGT	
E2Fa 308-341	CGGGATCCATGGTCAAAGCTCCTCATGG	GAAGGCCTAATAGGTCCCATTGTACT	
AKIN10 K48M	ATAAGGTTGCTATCATGATCCTCAATCGTCG	CGACGATTGAGGATCATGATAGCAACCTTAT	Site specific mutagenesis
E2Fa S93A	GTTGTTAGATCTCCTGCACGAAAGAGAAAGG	CCTTTCTCTTTCGTGCAGGAGATCTAACAAC	
E2Fa T99A	CGAAAGAGAAAGGCGGCAATGGATATGGTTG	CAACCATATCCATTGCCGCCTTTCTCTTTCG	
E2Fa S170A	AGTTGTCGTTATGACGCTTCTTTAGGTCTCCT	AGGAGACCTAAAGAAGCGTCATAACGACAACT	
E2Fa S278A	AGATTAAGAGACCTGGCCGAAAATGAAAAGAA	TTCTTTTCATTTTCGGCCAGGTCTCTTAATCT	
E2Fa S427A	CTTAAGATTACTCCCGCTGATGTTGAAAATG	CATTTTCAACATCAGCGGGAGTAATCTTAAG	
E2Fa S454A	ATTTGGAAAACTGACGCTGGTATCGATTGGG	CCCAATCGATACCAGCGTCAGTTTTCCAAAT	
E2Fa T314A	AAAGCTCCTCATGGCGCAACTTTGGAAGTGCCTG	CAGGCACTTCCAAAGTTGCGCCATGAGGAGCTTT	
E2Fa T315A	GCTCCTCATGGCACAGCTTTGGAAGTGCCTG	CAGGCACTTCCAAAGCTGTGCCATGAGGAGC	
E2Fa T314AT315A	AAAGCTCCTCATGGCGCAGCTTTGGAAGTGCCTG	CAGGCACTTCCAAAGCTGCGCCATGAGGAGCTTT	
E2Fa T315D	GCTCCTCATGGCACAGATTTGGAAGTGCCTGA	TCAGGCACTTCCAAATCTGTGCCATGAGGAGC	
E2Fa T314AT315D	AAAGCTCCTCATGGCGCAGATTTGGAAGTGCCTGA	TCAGGCACTTCCAAATCTGCGCCATGAGGAGCTTT	
E2Fa T314DT315D	AAAGCTCCTCATGGCGATGATTTGGAAGTGCCTGA	TCAGGCACTTCCAAATCATCGCCATGAGGAGCTTT	
E2Fa S338A	AGGATCATTCTTAGAGCTACAATGGGACCTAT	ATAGGTCCCATTGTAGCTCTAAGAATGATCCT	
E2Fa T339A	ATCATTCTTAGAAGTGCAATGGGACCTATTG	CAATAGGTCCCATTGCACTTCTAAGAATGAT	
E2Fa S338AT339A	AGGATCATTCTTAGAGCTGCAATGGGACCTATTG	CAATAGGTCCCATTGCAGCTCTAAGAATGATCCT	
qRT-DIN1	CAGAGTCGGATCAGGAATGG	ATTTGACCGCTCTCACAACC	RT-PCR and RT-qPCR
qRT-DIN6	AACTTGTCGCAAGATCAAGG	GGAACACGTGCCTCTAGTCC	
qRT-E2Fa	AGGCCAAAGGAAACAAGTCAACTCC	TGCAGCTTTGTTTAGGTCCAGCATT	
qRT-E2Fb	GAGGAAAGCACCGAAAGAAACATGG	TGACTTCGCCTACCTCTGATCGAA	
qRT-ETG1	CCCACGCCTCCATTGTCTTATCC	GAACTGTGCGGCAATGTGATCATTC	
qRT-MCM3	CTTCGCCACAAGCGAGATTTTATCC	TGGCTGCGTCACAAAATGACTG	
qRT-MCM5	CAATTCGCCAGCCTTATATCCGAGT	GGAGCGATCTTGGTGCAAATGTTC	
qRT-ORC2	TGGGTGGGGCGAGTAAGCGT	AGGCCAAAGCCACACCTGAGC	
qRT-PCNA1	CCTGATGCTGAGTACCACTCAATCG	TGAGCACAATGTTAGCGGTTCCA	
qRT-ELF4a	TCATAGATCTGGTCCTTGAAAC	GGCAGTCTCTTCGTGCTGAC	
UBQ10	AGATCCAGGACAAGGAGGTATTC	CGCAGGACCAAGTGAAGAGTAG	
NOS-T		GCAAGACCGGCAACAGGATT	
AKIN10K48M BglII	AGAATGGATGGATCAGGCAC	GATTTTGCGACGATTGAAGATC	dCAPS

Reference

Porra R, Thompson WaA, Kriedemann P. 1989. Determination of accurate extinction coefficients and simultaneous equations for assaying chlorophylls a and b extracted with four different solvents: verification of the concentration of chlorophyll standards by atomic absorption spectroscopy. *Biochimica et Biophysica Acta (BBA)-Bioenergetics* **975**(3): 384-394.
